# Are hotspots and frequencies of heat waves changing over time? Exploring causes of heat waves in a tropical country

**DOI:** 10.1371/journal.pone.0300070

**Published:** 2024-05-22

**Authors:** Md. Mostafizar Rahman, Md. Abdul Mannan, Md. Sujahangir Kabir Sarkar, Muhammad Abul Kalam Mallik, Afruza Sultana, Md. Kamrul Islam, Mst Yeasmin Akter, Edris Alam, Abu Reza Md. Towfiqul Islam

**Affiliations:** 1 Bangladesh Meteorological Department, Dhaka, Bangladesh; 2 Department of Economics and Sociology, Patuakhali Science and Technology University, Patuakhali, Bangladesh; 3 Department of Civil and Environmental Engineering, College of Engineering, King Faisal University, AlAhsa, Saudi Arabia; 4 Department of Disaster Management, Begum Rokeya University, Rangpur, Bangladesh; 5 Department of Geography and Environmental Studies, University of Chittagong, Chittagong, Bangladesh; 6 Faculty of Resilience, Rabdan Academy, Abu Dhabi, United Arab Emirates; 7 Department of Development studies, Daffodil International University, Dhaka, Bangladesh; University 20 Aout 1955 skikda, Algeria, ALGERIA

## Abstract

Heat waves significantly impact people’s lives and livelihoods and are becoming very alarming and recognized as hot topics worldwide, including in Bangladesh. However, much less is understood regarding recent hotspots, the frequency of heat waves over time, and their underlying causes in Bangladesh. The objective of the study is to explore the current scenario and frequency of heat waves and their possible causes across Bangladesh. The Mann-Kendall and Sen’s slope techniques were used to determine seasonal and annual temperature trend patterns of heat wave frequencies. Daily maximum temperature datasets collected from the Bangladesh Meteorological Department (BMD) during 1991–2021 are applied. The frequency of days with Tmax≥ 36°C as the threshold was used to compute different types of heat waves based on the BMD’s operational definition. The results show that the mild heat wave (MHW) days followed the subsequent hotspot order: Rajshahi (103) > Chuadanga (79), Ishurdi (60), and Jessore (58), respectively. The frequency of days with Tmax≥36°C was persistence for many days in 2014, especially in the western part of Bangladesh compared to other parts. Similarly, the heat waves condition shown its deadliest event by increasing more days in 2021. The highest increasing trend was identified at the Patuakhali site, with a rate of 0.516 days/year, while the highest decreasing trend was noticed at the Chuadanga site, with a rate of -0.588 days/year. The frequency of days (Tmax≥36°C) is an increasing trend in the south-western part of Bangladesh. The synoptic condition in and around Bangladesh demonstrates that the entrance of heat waves in Bangladesh is due to the advection of higher temperatures from the south/southwest of the Bay of Bengal. The outcomes will guide the national appraisal of heatwave effects, shedding light on the primary causes of definite heatwave phenomena, which are crucial for developing practical adaptation tools.

## 1. Introduction

According to the IPCC (2018) report, severe weather events caused by global warming of 1°C over pre-industrial levels are affecting society and ecosystems [1]. It is a matter of concern that heat waves harm human health and the agricultural and energy sectors [2–6]. A recent study found that extreme heat triggers yearly fatalities [7]. Heat waves’ characteristics are duration, frequency, severity, and geographic extent [8]. However, several researches have been done on the frequency and geographic area, particularly in Bangladesh [9]. Bangladesh is very vulnerable to various kinds of meteorological phenomena, such as cold waves, heat waves, heavy downpours, thunderstorms, cyclones, etc., due to its location. Out of these, the heat wave is one of the most recent to hit the table because of its striking adverse impact on socio-economic activities and other health-related hazards across the country during the pre-monsoon (March to May) and monsoon season (June to September). In terms of meteorology, the seasons in Bangladesh have been classified into four classes: cool and dry winter (December to February), warm pre-monsoon summer (March to May), the rainy season (June to September), and post-monsoon autumn (October to November). Lubna et al. (2020) noted that continental heating that produces a low-pressure monsoon trough increases throughout the pre-monsoon period, and this low-pressure system is anchored by the Tibetan Plateau, which is known as the third pole of the world, and the Himalayas, extending the heating throughout the troposphere [10]. The persistence of heat waves for some consecutive days has a disastrous impact on human and animal health, economics, livelihood, and the ecosystem. The combination of extreme heat and high humidity may cause discomfort, heat stroke, or even death to humans and animals.

Various authors have studied heat wave-related incidences extensively [11–14]. In 2015, four of the ten deadliest natural disasters occurred by heat waves; in terms of mortality, South Asian heat waves ranked third and fourth [15]. It has been studied that Bangladesh is dangerously threatened by climate change, and heat waves will increase in frequency and intensity in the near future [16]. It has been explored in a study that Rajshahi has the highest mean frequency of Tmax ≥36°C in May. In contrast, Dinajpur and Rangpur have the maximum mean frequency of Tmax ≥36°C in April [17]. In the context of Bangladesh, several works have been accomplished on defining, predicting, and evaluating the heat wave status [9, 18–21], though lots of studies have been done in India [22–29]. In the present study, monthly and annual frequencies of maximum temperature (Tmax≥36°C) have been regarded to explore heat waves situation. In Bangladesh, heat wave is classified into four categories: a mild heat wave denotes 36 to 38°C, a moderate heat wave represents 38.1 to 40°C, a severe heat wave signifies 40.1 to 42°C, and an extreme heat wave denotes >42°C [17].

The long-term climatology and the frequency of heat waves have been studied during 1961–2010 in India [23]. In a study, it has been observed that heat waves have increased and may continue to increase under future warming [30]. Heat waves and warm spells have been studied in India, as observed from a different aspect framework. The study ran during three time periods (1951–2013, 1981–2013, and 1998–2013) [25]. This region is experiencing an annual temperature rise of 0.4–0.6°C during the period 1989–2019, which is very alarming for us because of its adverse effects [31]. However, there is a lack of research on the recent heat wave hotspots in Bangladesh’s climatic condition scenario. Therefore, knowledge and understanding of current systems and the trend of heat waves are crucial for developing HEWS in Bangladesh. The stations’ missing data were below 2% from 1991 to 2021. Missing data were occupied by existing records for the particular days from the adjacent site.

Recent frequencies and potential causes of heat waves were not fully reported to the esteemed research community and academics regarding heat wave-related studies in undeveloped countries. For example, Nissan et al. [32] conducted a case study based on the model and found that the future changes in heat wave seasonality in Bangladesh comprise the modeled seasonality of temperature and heat wave frequency in the past. Tawsif et al. [33] analyzed the temperature time series data from 1984–2018 and concluded that the period of heat waves is increasing daily. Imran et al. [34] showed positive warming trends in the mean maximum and minimum temperatures. However, a few earlier studies have focused on the trends of different types of heat waves and causes in Bangladesh [32, 35]. Therefore, an apparent understanding of the underlying reasons for heat waves is required to aid in formulating site-specific mitigation and adaptation measures.

Heat wave extent, frequency, and intensity have previously augmented in many parts of the globe [36], and this trend is anticipated to increase in the forthcoming period [37]. Addressing climate change impact at the regional and national levels, particularly in areas of complicated hydrogeographic settings, including Bangladesh, requires suitable quality datasets. Thus, understanding and managing the increasingly intense heat waves occurring regionally and globally possibly one of the most remarkable climate extremes in the future requires substantial investigations.

Bangladesh is a climate change ‘hotspot’ [38] due to its position at the line between temperate and semi-humid climates. This makes the country mostly vulnerable to changes in the general circulation pattern [39, 40]. Only a few studies have investigated the impact of heat stress in the country, concentrating primarily on local and regional scales [20]. The assessment of the frequencies and variabilities of heat waves and their probable causes has hardly been conducted for the country. To address these issues, our study aims to (i) investigate the recent scenarios and frequencies and variabilities of Tmax that are responsible for producing heat waves; (ii) demonstrate the hotspots of heat waves across Bangladesh and the advection of heat waves in Bangladesh; (iii) explore the possible causes of heat waves to some extents in Bangladesh. The novel aspect of this research is that it identifies hotspots and frequencies of heat waves over time across Bangladesh and its possible cause of heat wave changes. Further, this study will offer scientific justification for government and non-government public health strategies that target future behavioral and technical adaptations to climate change.

## 2. Data and methodology

### 2.1 Study area

**Fig 1:**
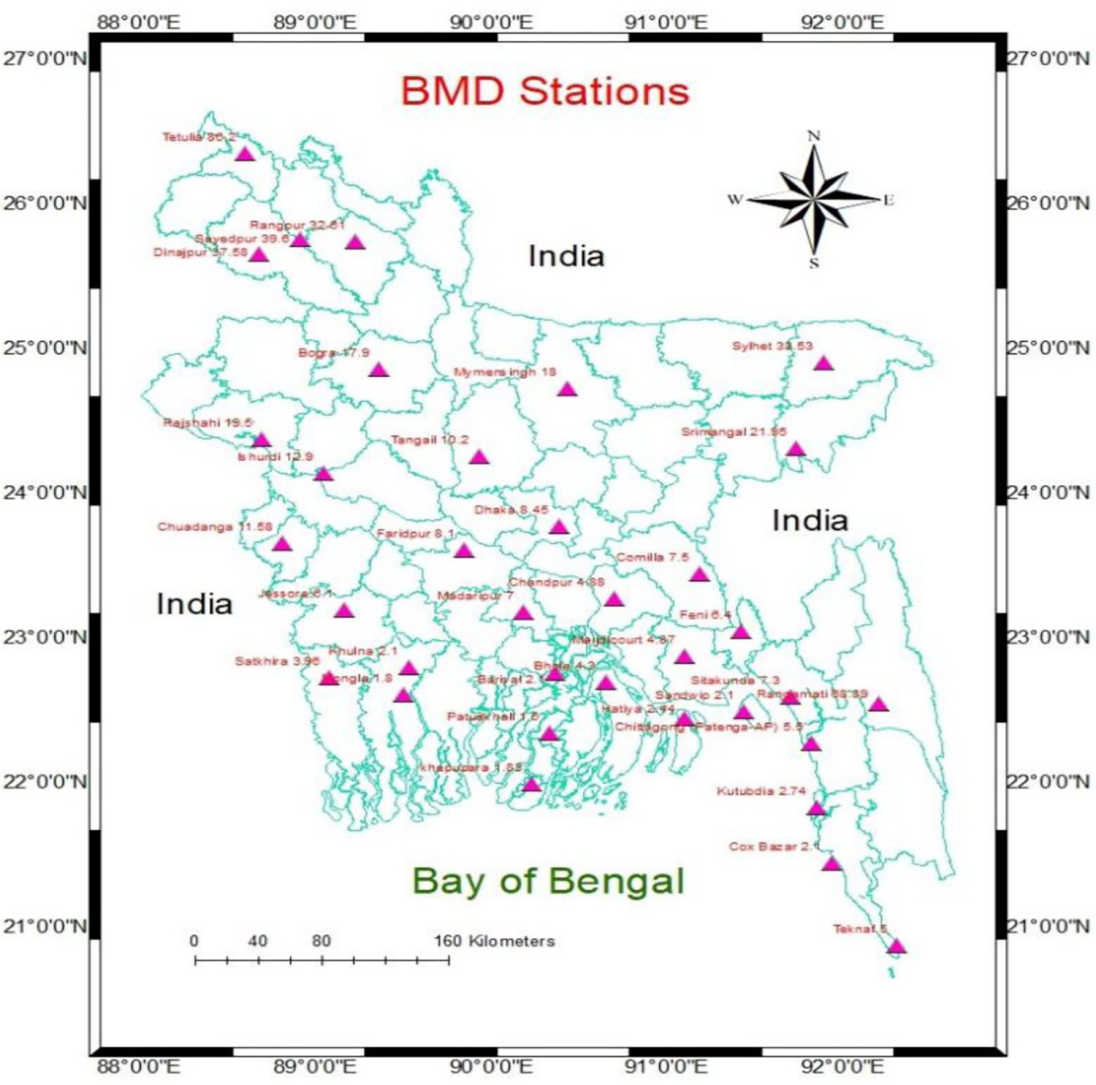
Location of the selected Bangladesh meteorological station.

Bangladesh is a country in South Asia, located in the delta of the Padma (Ganga) and Jamuna (Brahmaputra) rivers in the northeastern part of the Indian subcontinent. Bangladesh is bordered by the Indian states of West Bengal to the west and north, Assam to the north, Meghalaya to the north and northeast, and Tripura and Mizoram to the eastern part. To the southeast, it shares a boundary with Myanmar (Burma). Most of Bangladesh’s topography is low lying area, except for some highland areas in the northeast and southeast regions. The Bay of Bengal is situated in the southern part of Bangladesh. The study has depicted 32 meteorological stations across the country shown in **Fig 1.**

### 2.2 Data sources and quality control

In our study, we used daily, monthly, annual maximum temperature datasets of 32 meteorological stations across Bangladesh for the period of 1991–2021, collected from Bangladesh Meteorological Department (BMD) **(Table 1).** Among these stations, some of the meteorological stations are newly set up and these stations do not have long-range data records. This is why, 32 stations were selected for the study purpose and these sites encompass every part of the country. The missing data of these sites were below 2% during the period from 1991to 2021. Missing data for each site were occupied by existing records for the particular days from the adjacent site. The maximum temperature of each year during 1991–2021 was separated in different categories such as mild heat waves, moderate heat waves, and severe heat waves.

**Table 1:** The meteorological stations across Bangladesh.

Sn	ID_WMO	Name	Longitude	Latitude	Elevation
1	41858	Sayedpur	88.92	25.75	39.6
2	41859	Rangpur	89.23	25.73	32.61
3	41863	Dinajpur	88.68	25.65	37.58
4	41883	Bogra	89.37	24.85	17.9
5	41886	Mymensingh	90.43	24.72	18
6	41891	Sylhet	91.88	24.9	33.53
7	41895	Rajshahi	88.7	24.37	19.5
8	41907	Ishurdi	89.05	24.13	12.9
9	41909	Tangail	89.93	24.25	10.2
10	4198	Srimangal	91.73	24.3	21.95
11	41923	Dhaka	90.38	23.77	8.45
12	41926	Chuadanga	88.82	23.65	11.58
13	41929	Faridpur	89.85	23.6	8.1
14	41933	Comilla	91.18	23.43	7.5
8	41936	Jessore	89.17	23.18	6.1
16	41939	Madaripur	90.18	23.17	7
17	41941	Chandpur	90.7	23.27	4.88
18	41943	Feni	91.42	23.03	6.4
19	41946	Satkhira	89.08	22.72	3.96
20	41947	Khulna	89.53	22.78	2.1
21	41950	Barisal	90.37	22.75	2.1
22	41951	Bhola	90.65	22.68	4.3
23	41953	Maijdicourt	91.1	22.87	4.87
24	41958	Mongla	89.5	22.6	1.8
25	41960	Patuakhali	90.33	22.33	1.5
26	41965	Sitakunda	91.7	22.58	7.3
27	41966	Rangamati	92.2	22.53	68.89
28	41977	Chittagong	91.82	22.35	33.2
29	41984	khepupara	90.23	21.98	1.83
30	41989	Kutubdia	91.85	21.82	2.74
31	41992	Cox Bazar	91.93	21.43	2.1
32	41998	Teknaf	92.3	20.87	5

National Centre for environmental prediction (NCEP) reanalysis data are used for observing atmospheric condition when more active heat wave spell swept over Bangladesh during 20–25 April 2014 and during month of April in the year of 2021(https://psl.noaa.gov/data/gridded/data.ncep.reanalysis). Surface wind and relative humidity (RH), at different level are conducted to validate of atmospheric condition which are directly linked with heat waves over Bangladesh and the adjoining area. During the month of April 2014 and 2021, some severe and very severe incidents of heat waves occur. Wind flows near the surface are considered to validate atmospheric conditions which are associated with heat waves over Bangladesh and the neighborhood. In 2014, the frequency of days with Tmax≥36°C was persistence for many days, especially in the western part of Bangladesh compared to other parts. Likewise, the heat waves condition revealed its deadliest characteristics by increasing and prevailing more days in 2021. That is why in our study took these two years in our consideration to explore heat wave situation in Bangladesh.

### 2.3 Climatology of heat waves in Bangladesh

The Intergovernmental Panel on Climate Change has recognized the Bangladesh as one of the utmost vulnerable to climate change [1]. Bangladesh has a subtropical monsoon climate characterized by wide seasonal variations in rainfall, moderately warm temperatures, and high humidity, cold and dry weather in the winter, and a warm, humid summer [41]. The climate of Bangladesh has some unique criteria. Bangladesh experiences four distinct seasons: pre-monsoon (March-May), monsoon (Jun-Sept), post-monsoon (Oct-Nov), and winter (Dec-Feb) [40]. The monsoon season receives the majority of the annual rainfall, whereas the winter season is marked by little rainfall. The country’s northwest and northeast receive varying amounts of rainfall, ranging from 1200 to 4300 mm [34]. Nonetheless, the nation’s average annual temperature is around 25°C, with April being the warmest month. During the rainy season, on average 2100 mm of rainfall occurs per year, and December is the driest month with a mean of 5 mm [20]. Throughout the pre-monsoon period, a low-pressure system and an east-west extended trough prevailed over the northern part of Bangladesh. A strong equatorial pressure gradient is produced, accelerating the monsoon’s arrival in the southeastern part of Bangladesh in the first week of June. Later on, the monsoon moves toward the northwest part of Bangladesh. After persisting up to September, monsoon retreats from the northwestern region in late September and finally from the southwest part in early October [42]. During this season, the highest temperatures are usually recorded in the western part, and all parts of the country receive rainfall, but the northeastern part records the maximum rainfall [42]. Most of the heat waves occur due to high diurnal temperature range and high humidity persisting across the country during April to June [43]. The country is divided into seven separate climate zones: the northern, north-central, north-eastern, south-western, western, and south-eastern zones. Bangladesh’s climate is predicted to gradually warm up and experience changes in rainfall patterns, which could have an impact on the country’s agriculture, public health, and water supply [40]. The pre-monsoon season is treated as a transition period because this period has both the experience of wind circulation that initiates in the northern part of Bangladesh and the southeasterly summer monsoon. This region is struck by a tropical cyclone in pre-monsoon season and reaches its apex number in November. In the winter season, the northern part of the country, experiences the lowest temperature throughout the year. The hot, dry northwesterly air mass is separated from the moist, southerly flow arriving from the Bay of Bengal by a "zone of discontinuity" [38]. Thunderstorms and convective rains are seen in this season, and these are developed because of the southerly winds that carry huge moisture content [42]. Despite these climatic events, due to the lack of accurate updated temperature datasets, little research has been done on the hotspots and frequencies of heat waves over time across Bangladesh and its possible causes.

### 2.4 Mann Kendall (Mk) trend test

The non-parametric Mann-Kendall (MK) test was used for tendencies classifying in climatologic and weather data in time series analysis. The trend analysis plays an important role that uses historical data of various meteorological parameters. To eliminate auto-correlation, the pre-whitening procedure initially applied to the time series of heatwave attributes. The Mann-Kendall trend test was employed to determine whether one existed. With a 95% confidence interval, the results were regarded as significant. Here, there are two benefits of using MK experiment. (a) It is a non-parametric test where data do not necessary to be normally distributed. (b) The trial has little sensitivity to unexpected due to inhomogeneous time series analysis [44]. The MK test statistic (S) is given by:

$$S=\sum_{i=1}^{n-1}\sum_{j=i+1}^{n}sign(T_{j}-T_{i}),$$ (1)

$$Sign(T_{j}-T_{i})=\left\{ \begin{array}{c} 1\;if\;T_{j}-T_{i}>0\\ 0\;if\;T_{j}-T_{i}=0\\ -1\;if\;T_{j}-T_{i}<0. \end{array}\right.$$ (2)

Where, *T*_*j*_ and *T*_*i*_ are the yearly values in years j and i, j > i respectively.

If n < 10, the value of |*S*| is shares straight to the theoretical distribution of S derived MK. The two tailed experiment is used. At definite likelihood level *H*_0_ is rejected in favor of *H*_1_ if the original value of S equals or exceeds a certain value *S*_*α*/2_, where *S*_*α*/2_ is the lowest S which has the possibility less than α/2 to execute in case of no trend. A positive (negative) value of S designates an upward (downward) trend. For n ≥ 10, the statistic S is almost normally distributed with the mean and variance as follows:

E(S)=0. (3)

The σ^2^ for the S statistic is expressed by:

$$\sigma^{2}=\frac{n(n-1)(2n+5)-\sum t_{i}(i)(i-1)(2i+5)}{18}.$$ (4)

Where *t*_*i*_ represents the number of ties to extent i. The synopsis term in the numerator is used only if the data series contains tied values. The standard test statistic *Z*_*s*_ is considered as bellows:

$$Z_{s}=\left\{ \begin{array}{c} \frac{S-1}{\sigma }\;for\;S>0\\ 0\;for\;S=0\\ \frac{S+1}{\sigma }\;for\;S<0. \end{array}\right.$$ (5)

The test statistic *Z*_*s*_ is used a quantity of meaning of trend. In detail, this experiment statistic is used to experiment the null hypothesis, *H*_0_. If |*Z*_*s*_| is greater than *Z*_*α*/2_, where *α* denotes the special implication level (e.g., 5% with *Z*_0.025_ = 1.96) then the null hypothesis is inacceptable suggesting that the trend is important.

### 2.5 Sen’s Slope estimator test

Non-parametric Sen Slope estimator technique was used to assessment the degree of tendencies in the data at time series analysis. Theil-Sen’s estimator was utilized to assess the trend’s magnitude. The gradient of “n” pairs of statistics can be first projected by using the below equation:

$$\beta_{i}=\mathrm{Median}\left[\frac{X_{j}-X_{k}}{j-k}\right]\forall \;(k<j)$$ (6)

In this formula, *X*_*j*_ and *X*_*k*_ represent values statistics at time j and k, separately, and time j is after time k (k ≤ j). The median of “n” values of *β*_*i*_ is the Sen’s slope estimator experiment. A negative *β*_*i*_ value signifies a declining tendency; a positive *β*_*i*_ value signifies an accumulative tendency over time.

If “n” is an even number, then the slope Sen’s estimator is computed by using the following equation:

βmed=12(β[n2]+β[(n+2)2]) (7)

If “n” is an odd number, then the slope Sen’s estimator is calculated by using the below formula:

βmed=β[(n+1)2] (8)

Finally, *β*_*med*_ is verified by a two tailed experiment at 100(1-α) % assurance level, and the true gradient of monotonic tendency can be projected by using a nonparametric experiment [20].

### 2.6 Spatial analysis

Inverse distance weighting (IDW) is a deterministic model for interpolation with a known scattered set of points in the GIS technique. The assigned values to unknown sites are computed with a weighted of the values available at the known sites. The IDW method is a simple and easy to deploy interpolation model in comparison with the other interpolation technique in the case of limited dataset or irregular data pattern. In the meantime, it has reported that the IDW method is more beneficial when interpolating the distribution of the heat wave condition is changing and spreading over the country within a particular period. We used IDW method, because this method is more in agreement with the real-world research requirements.

## 3. Results

### 3.1 Seasonal and annual frequencies of days of T_max_ ≥36°с and trend across Bangladesh

As heat waves condition prevail when the temperature becomes more or equal 36°C, and the people in this region feel uneasy and discomfort, and conditions like heat stress occur, so in this study, monthly and annual frequencies Tmax≥36°C have been calculated during the period 1991–2021. The pre-monsoon (March, April, and May) and the monsoon (June to September) are the targeted periods under study. Though the main focus is the pre-monsoon season, when extreme heat waves are found across Bangladesh and the surroundings, intense heat waves also invade the monsoon season. Accordingly, MHW, MoHW, and SHW of 32 stations (under what we considered) across Bangladesh are analyzed. To explore the reasons for extreme heat waves in 2014, conditions of some atmospheric phenomena have been analyzed.

Analysis of maximum temperature Tmax ≥ 36°C has been conducted in two parts: one is the seasonal (Pre-monsoon and monsoon) frequencies of days, and another one is the annual frequencies of days for the period 1991–2021. Pre-monsoon and monsoon seasons contribute mainly to the annual frequencies of days with Tmax ≥ 36°C throughout the study.

The trend analysis shows a significant increasing trend of annual average day frequency (0.27 per year) of Tmax≥36°C. The rising trend is found in the discussed period (Pre-monsoon, Monsoon, and Annual) with different significance levels except in pre-monsoon when the trend is not found significant (Table 2). The maximum frequency occurred in 2019 in the monsoon season, whereas in the pre-monsoon, the maximum frequency was discovered in 2014. The trend is high in the monsoon season (0.18 per year). The trend analysis of the monthly frequency of days shows that all months show an increasing trend except March (Table 3). The highest trend is observed in both May (2.78) and June (2.56); the less increasing trend is found in April and July-September. The trend is significant from June to September and is insignificant during pre-monsoon.

**Table 2:** The trends in annual and seasonal average days of frequency (Tmax≥36°C).

Annual/seasonal	Trend (Days/Year)	*p*<
Pre-monsoon	0.11	0.561
Monsoon	0.18	0.001
Annual	0.27	0.044

Here ‘p’ indicates the probability determining the significance level and p<0.05 means 95% confidence level.

**Table 3:** Monthly trends in days of frequency (Tmax≥36°C).

Month	Trend	*p*<
March	-0.01	0.658
April	0.46	0.852
May	2.78	0.102
June	2.56	0.009
July	0.99	0.001
August	0.85	0.010
September	1.10	0.001

Here ‘p’ indicates the probability determining the significance level in where p<0.05 means 95% confidence level.

**Fig 2:**
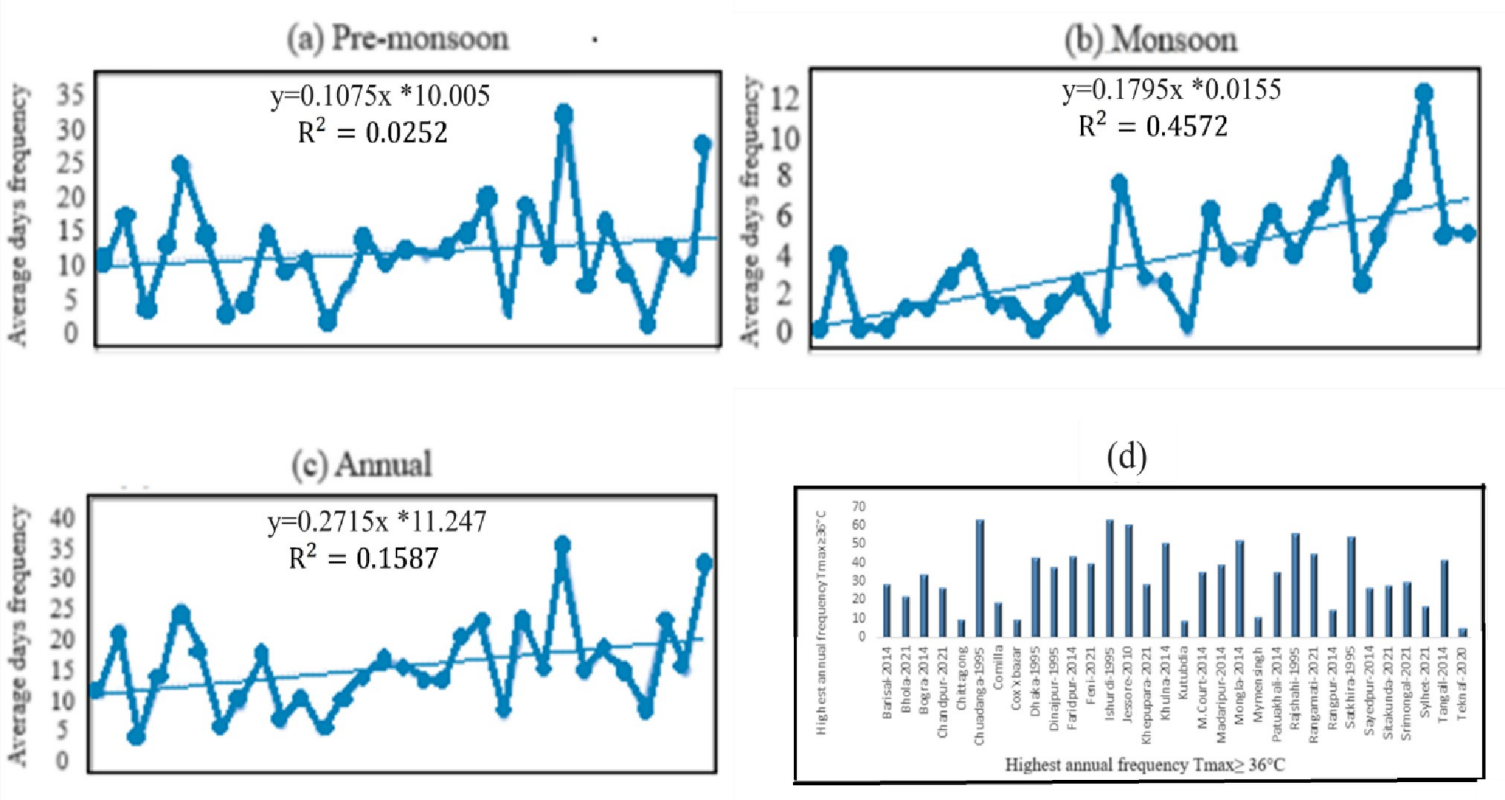
Frequency of days of T_max_ ≥ 36°C.

Fig 2 represents the frequencies of days with T_max_ ≥ 36°C for the period 1991–2021 and found that highest frequencies occurred in 2014. The maximum highest frequencies of days with T_max_ ≥ 36°с in the pre-monsoon season are 62, 62, 60, 55, and 55 days at Chuadanga in 1995, Ishurdi in 1995, Jessore in 2010, Rajshahi in 1995 and Khulna in 2014 respectively. But most of the stations were affected by heat waves condition throughout the country.

### 3.2 Annual frequencies of days with T_max_ ≥ 36°C in divisional stations

**Fig 3:**
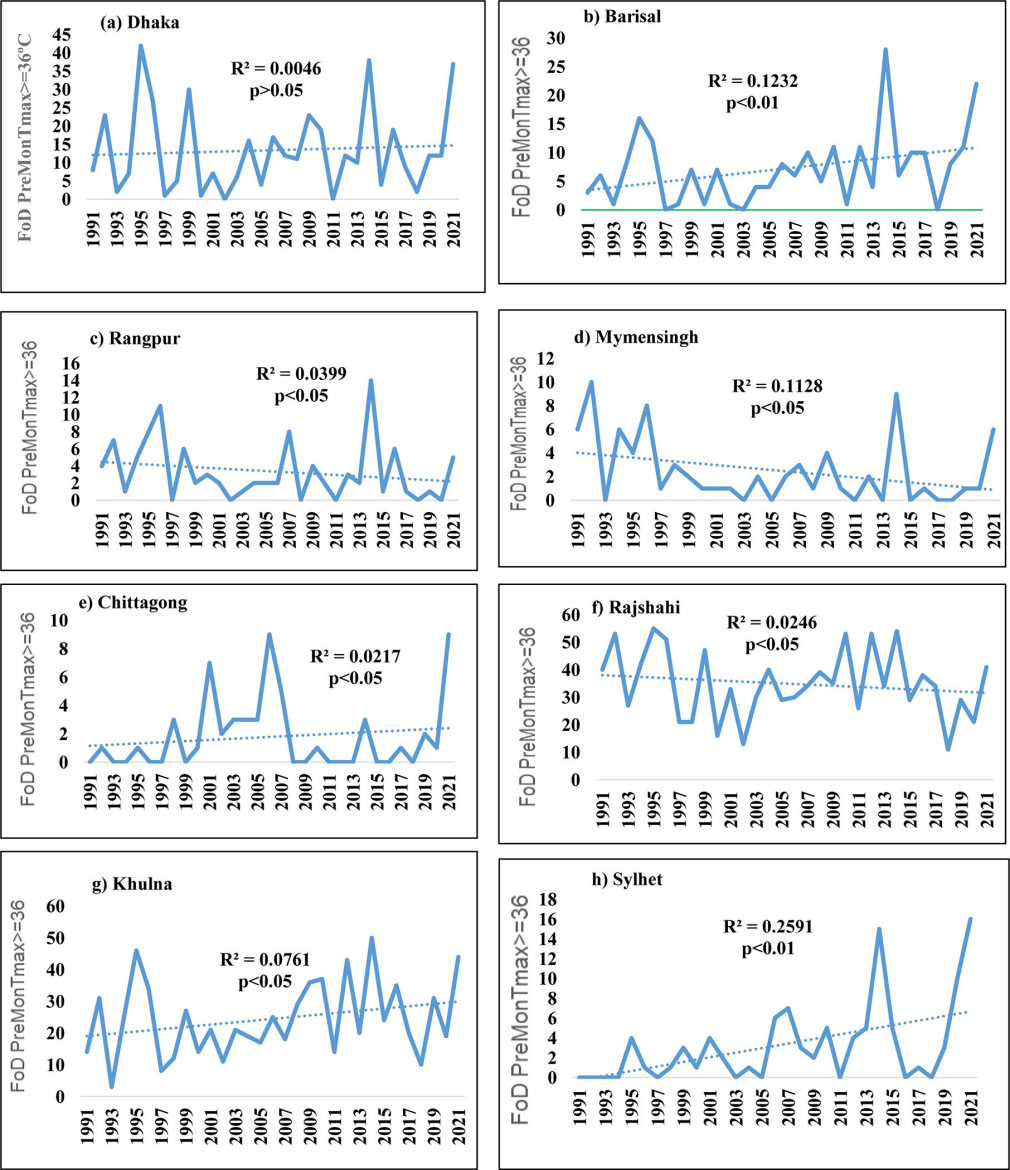
Trend and variability in annual frequency of days (FoD) with T_max_ ≥ 36°с at (a) Dhaka, (b) Barisal, (c) Rangpur, (d) Mymensingh, (e) Chittagong, (f) Rajshahi, (g) Khulna and (h) Sylhet during the pre-monsoon season of 1991–2021.

The trend and variability of frequency of days with Tmax ≥ 36°C at the divisional stations of BMD are given in Fig 3(A)–3(H). Figures show that in the Pre-monsoon period, the frequency of days with Tmax ≥ 36°C has an increasing trend at Dhaka, Chittagong, Barisal, Khulna, and Sylhet divisions. The rates of increament in the frequencies of days in the divisional stations are 0.087, 0.042, 0.257, 0.362, and 0.232 day/year respectively. The frequency of days with Tmax ≥ 36°C has a decreasing trend at Mymensingh, Rajshahi and Rangpur, and the rates are -0.105, -0.214 and -0.076 day/year respectively.

### 3.3 Frequency of days of mild, moderate, and severe heat waves

**Fig 4:**
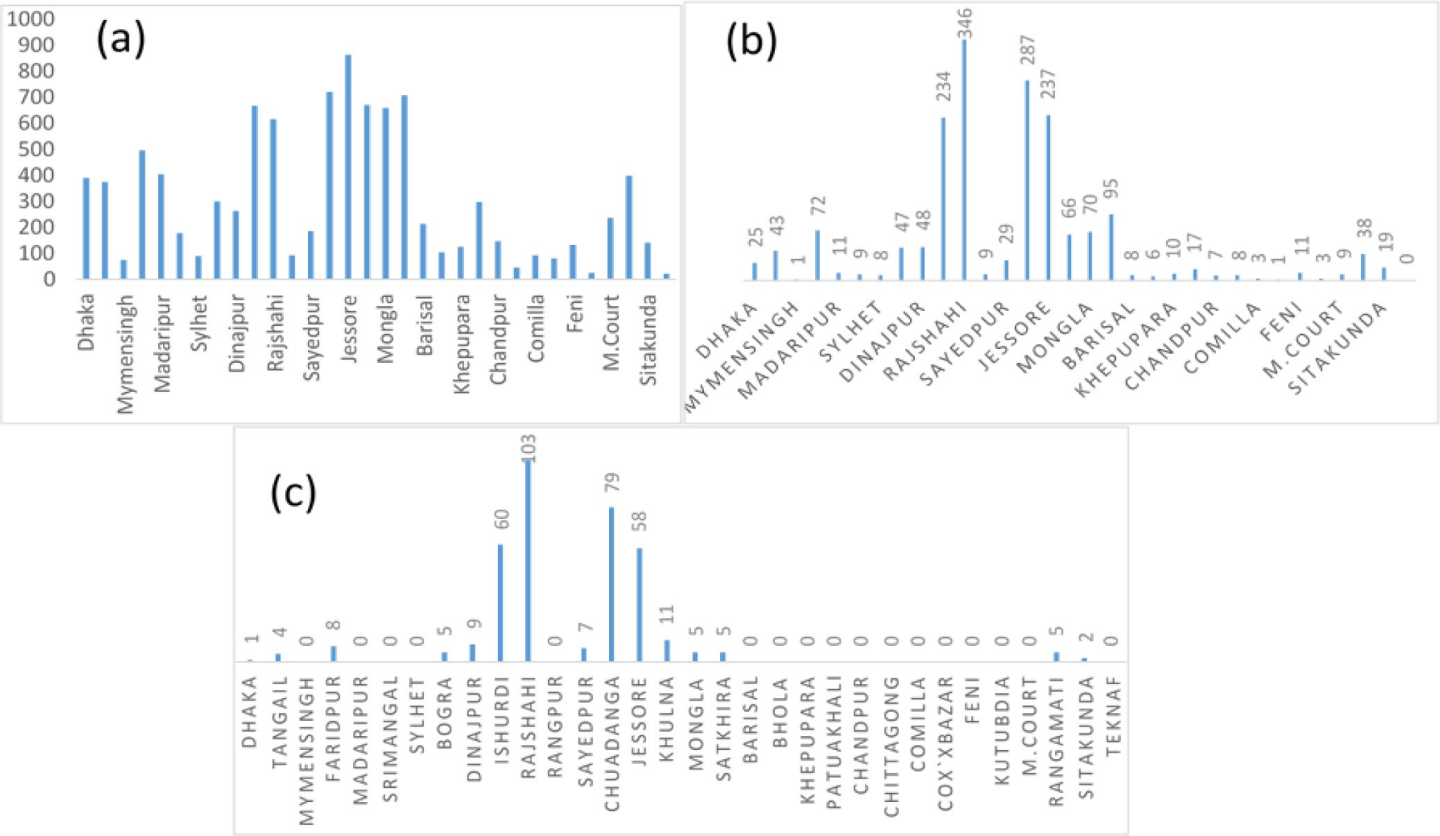
a) Highest frequency of days of MHW days b) Highest frequency of days of MoHW days c) Highest frequency of days of SHW days in pre-monsoon during 1991–2021.

Fig 4(A) shows the variation of frequency of days of mild heat wave (MHW) for the duration of 1991–2021 in 32 stations during Pre-monsoon across Bangladesh. It shows that the highest days of MHW found in Jessore which is 863 and second highest days found in Chuadanga 721 days then it is followed by Satkhira 708 days. The lowest frequencies of days were found in Teknaf, Kutubdia, Mymensingh, and most of the coastal districts.

Fig 4(B) shows the variation of frequency of days of moderate heat wave for the duration 1991–2021 in 32 stations during Pre-monsoon across Bangladesh. It shows that highest days of MoHW found in Rajshahi which is 346 and second highest days found in Chuadanga 287 days then it is followed by Jessore 237 days. The lowest frequencies of days were found in, Kutubdia, and most of the coastal districts. No MoHW hits Teknaf and negligible amount found in Mymensingh, Madaripur, Srimongol, Sylhet, Rangpur, and most of the coastal districts of Bangladesh.

Fig 4(C) shows the variation of frequency of days of severe heat wave for the duration 1991–2021 in 32 stations during Pre-monsoon across Bangladesh. It shows that highest days of SHW found in Rajshahi which is 103 and second highest days found in Chuadanga 79 days then it is followed by Ishurdi 60, and Jessore 58 days. The lowest frequencies of days were found in Kutubdia, and most of the coastal districts. No SHW hits Teknaf and negligible amount found in Mymensingh, Madaripur, Srimongol, Sylhet, Rangpur, Barisal, Khepupara, Patuakhali, Chandpur, Chittagong, Comilla, Cox’sbazar, Feni, Kutubdia and most of the coastal districts of Bangladesh.

Table 4 shows the seasonal trends and variability of the frequency of days with T_max_≥36°с in Bangladesh.

**Table 4:** Seasonal trends and variability of the frequency of days with T_max_≥36°с.

Stations		Seasonal trends and variability (Pre-monsoon)			
	Day/Year	R^2^	Tau-value	Sen`s slope	P-value
Dhaka	0.086	0.005	0.079	0.143	0.551
Tangail	0.161	0.024	0.131	0.227	0.316
Mymensingh	0.105	0.113	-0.277	-0.077	0.041**
Faridpur	0.066	0.003	0.100	0.200	0.444
Madaripur	0.134	0.017	0.146	0.214	0.261
Srimangal	0.109	0.019	0.081	0.000	0.548
Sylhet	0.231	0.259	0.371	0.166	0.006***
Bogra	-0.88	0.027	-0.121	-0.167	0.357
Dinajpur	-0.38	0.107	-0.231	-0.285	0.074*
Ishurdi	0.205	0.017	-0.084	-0.200	0.518
Rajshahi	-0.213	0.025	-0.089	-0.133	0.496
Rangpur	-0.076	0.040	-0.205	-0.077	0.125
Sayedpur	-0.089	0.016	-0.108	-0.100	0.412
Chuadanga	-0.558	0.116	-0.217	-0.611	0.092*
Jessore	-0.005	0.000	-0.0347	-0.080	0.798
Khulna	0.362	0.076	0.193	0.428	0.134
Mongla	0.488	0.140	0.299	0.550	0.021**
Satkhira	-0.091	0.005	0.008	0.000	0.051*
Feni	0.307	0.136	0.22	0.091	0.100*
Bhola	0.203	0.140	0.212	0.095	0.116
Barisal	0.247	0.123	0.249	0.200	0.058*
Kutubdia	-0.011	0.003	-0.018	0.000	0.98
Rangamati	0.426	0.111	0.262	0.285	0.043**
M.Court	0.484	0.309	0.446	0.462	0.001***
Sitakunda	0.214	0.128	0.194	0.125	0.141
Khepupara	0.281	0.175	0.248	0.125	0.063*
Cox`xbazar	0.075	0.068	0.146	0.000	0.282
Chittagong	0.042	0.022	0.102	0.000	0.471
Comilla	0.093	0.038	-0.112	0.000	0.48
Chandpur	0.248	0.184	0.428	0.250	0.001***
Patuakhali	0.516	0.303	0.405	0.500	0.002***
Teknaf	-0.012	0.010	-0.183	0.000	0.206

*Significant at 90%, **Significant at 95%, ***Significant at 99%

The station-wise seasonal (pre-monsoon) trends are calculated and shown in Table 4. The table shows that 22 out of 32 exhibit an increasing trend, whereas 13 stations show a significant increase. The negative trends concentrate in some areas, namely the north-western part (Bogra, Rajshahi), northern part (Rangpur, Sayedpur), south-western region (Jessore, Chuadanga, Satkhira), southeastern region (Kutubdia, Teknaf). Almost similar trends were observed in Dhaka, Faridpur, and Cox’sbazar. It showed that a positive trend is very dominant throughout Bangladesh.

### 3.4 Variation of heat waves days in the most affected stations

**Fig 5:**
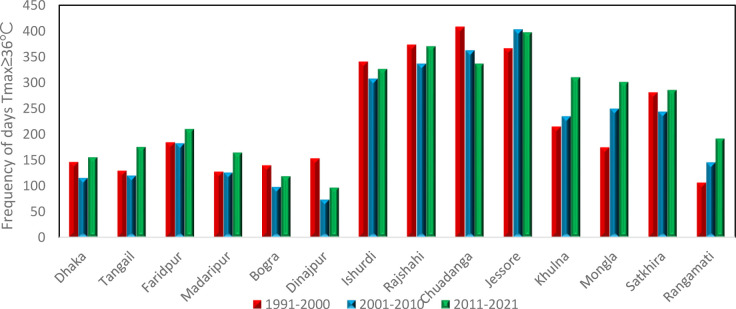
Frequency of heat wave days over fourteen different stations for decades D1, D2, and D3 for the period 1991–2021.

Fig 5 shows the variation of frequency of days of Tmax≥36°C for the three different decades, 1991–2000, 2001–2010, and 2011–2021, based on decadal variation. It shows that Dhaka, Bogra, Ishurdi, Rajshahi, and Satkhira follow the same pattern during all three decades 1991–2000, 2001–2010, and 2011–2021, namely D1(1991–2000), D2(2001–2010), and D3(2011–2021) respectively in which frequency of heat waves days Tmax≥36°C have increased. It also shows that Chuadanga experienced the highest frequency of heat waves (408) during D1, which decreases to 12 in D3 and is the highest in this decade among all fourteen stations. Jessore experienced the highest frequency of heat waves (403) during D2. Jessore experienced the highest frequency of heat waves during D2 (403) and D3 (397). In Mongla and Rangamati the trend is increasing regularly. Though Chuadanga experiences the highest frequency of heat wave days, the trend follows a decreasing pattern.

### 3.5 Decadal variation of mild heat wave days and moderate heat wave days in most affected stations during 1991–2021 during pre-monsoon

**Fig 6:**
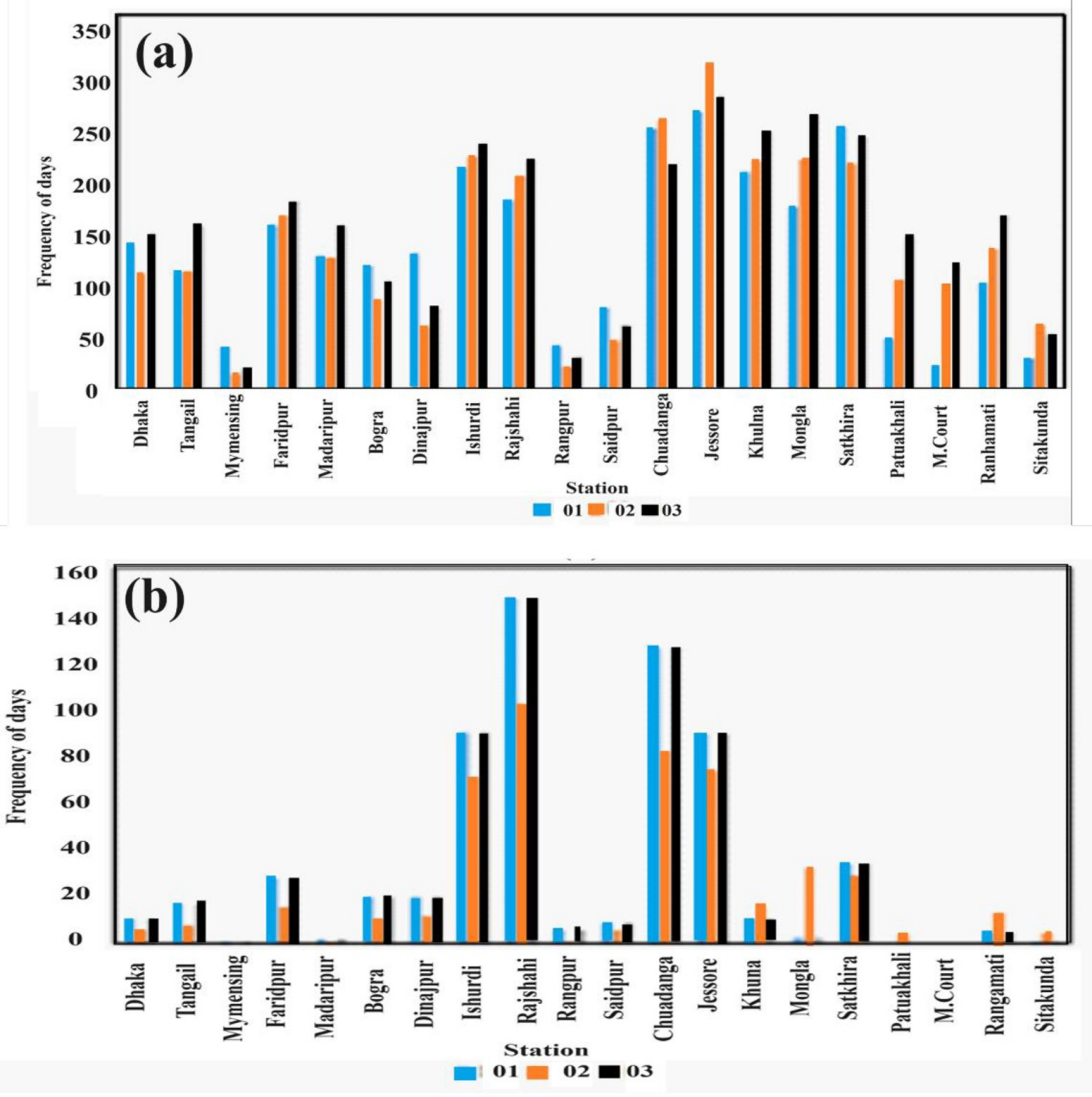
Frequency of a) MHW days b) MoHW days over fourteen different stations for decades D1, D2, and D3 during 1991–2021.

Fig 6(A) shows the frequency variation of MHW days for the three decades from 1991–2000, 2001–2010, and 2011–2021. It indicates that Faridpur, Khulna, Ishurdi, Rajshahi, Patuakhali, Rangamati, and Mongla follow the same pattern during all three decades, namely D1, D2, and D3 in where frequency of MHW days have increased gradually in an ascending order. It also shows that Jessore experienced the highest frequency of MHW days (304) during D2, which then decreases to 274 in D3 and is the highest in this decade among all fourteen stations. Jessore experienced the highest frequency of MHW days (259) during D1, the highest figure among all other stations. The most increasing trend was found in M. Court, which is 22 days in D1 and 97 days in D3. Fig 6(B) shows the variation of frequency of MoHW days for the three different decades during the 1991–2021. It shows that Tangail, Bogra, Ishurdi, Rajshahi, Jessore, Dinajpur and Satkhira follow the same pattern during all the three decades namely D1, D2, and D3 in which frequency of MoHW days was a decreasing trend in D2. It also shows that Rajshahi experienced highest frequency of heat waves (147) during D1 and D3. The second highest frequency of MoHW days was experienced by Jessore.

### 3.6 Monthly frequency of mild, moderate and severe heat wave events

**Fig 7:**
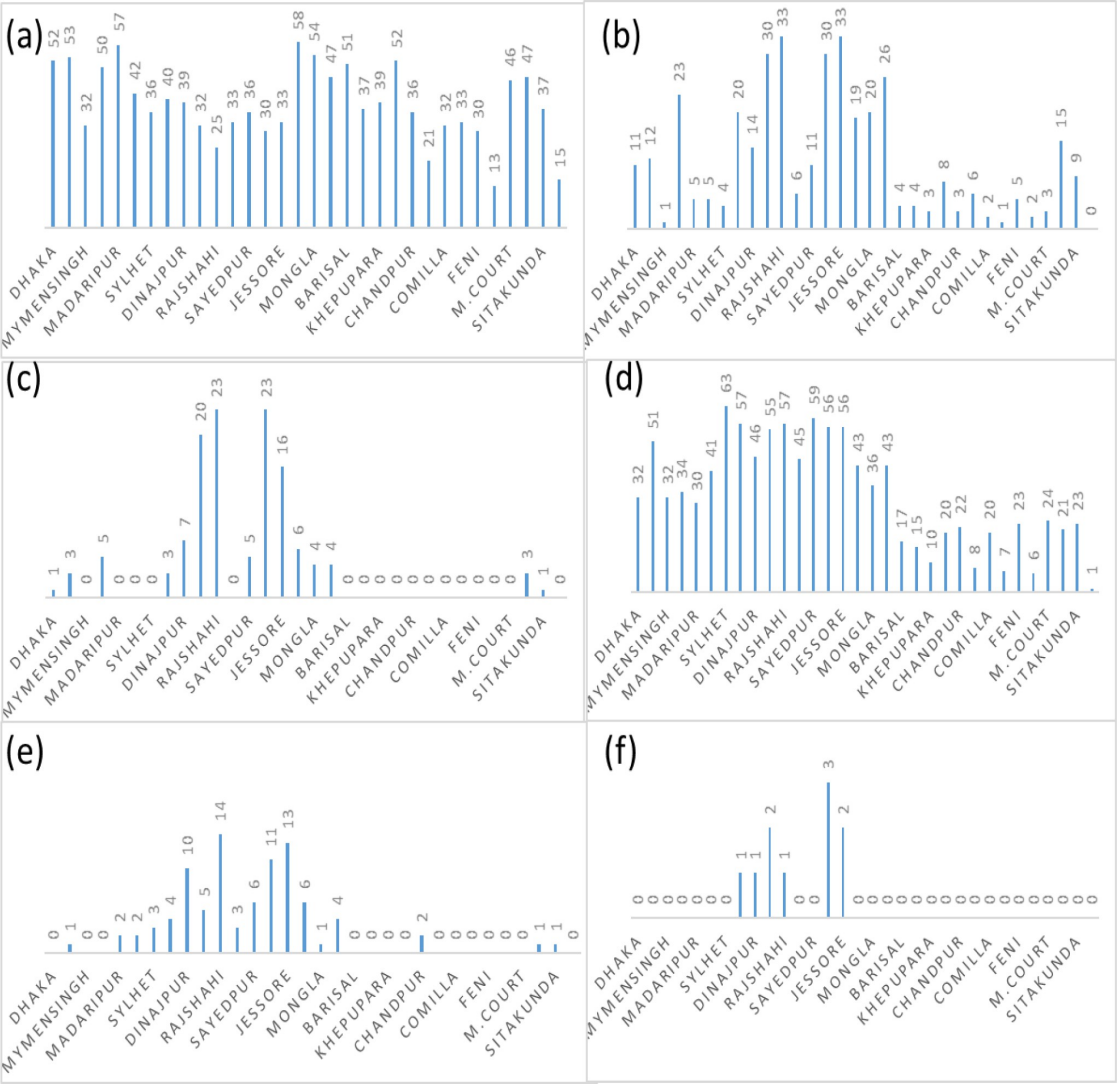
Monthly frequency of a) Mild heat waves days b) Moderate heat waves days c) Severe heat waves days during pre-monsoon and d) Mild heat waves days e) Moderate heat waves days f) Severe heat waves days during monsoon for the period 1991–2021.

The changing pattern and the trends of monthly frequencies of MHW (36–38°C) days for the duration of 1991–2021 are shown in Fig 7(A). The figure shows the southwestern parts of the country are very vulnerable as maximum frequency trends of MHW days hit this region. The highest frequencies of MHW days ranging 36–38°с are 58, 57, 54, 53, 52, and 51 days at Khulna in 1995, Madaripur in 1995, Mongla in 2010, Tangail in 1995, and Patuakhali in 2014, respectively in the pre-monsoon season.

The changing pattern and the trends of monthly frequencies of MoHW days ranging 38.1–40°C for the duration 1991–2021 are shown in Fig 7(B). The figure shows the southwestern parts of the country are very vulnerable as maximum frequency trends of MoHW days hit this region. The highest frequencies of days with MoHW are 33, 30, and 26 days at Rajshahi and Jessore in 1995, Ishurdi and Chuadanga in 1995, Satkhira in 2010 respectively, in the pre-monsoon season.

The spatial distributions of the trends of monthly frequency of SHW defined 40.1–42°C in the duration 1991–2021 are shown in Fig 12. shows maximum trends of the frequency of SHW 40.1–42°с over southwestern parts of the country. In the pre-monsoon season, the highest frequencies of days with SHW 40.1–42°с were 23, 20, and 16 days at Rajshahi and Chuadanga, Ishurdi, and Jessore in 1995 (Fig 7C).

In the study it is found, the highest frequencies of days with MHW 36.1–38°с are 63, 59, 57, 56, 55 and 51 days at Sylhet, Sayedpur, Rajshahi and Bogura, Jessore, and Chuadanga, Ishurdi, Tangail in 1995 respectively, in the monsoon season.

Fig 7(E) explores that in the monsoon season, the highest frequencies of days with MoHW38.1–40°с are 14, 13, and 11 days at Rajshahi, Jessore, and Chuadanga in 1995.

The highest frequencies of days with SHW 40.1–42°с are 3, 2 and 2 days at Chuadanga, Ishurdi, and Jessore in 1995, respectively, in the monsoon season (Fig 7F).

### 3.7 Heat wave in 2014 as a case study

**Fig 8:**
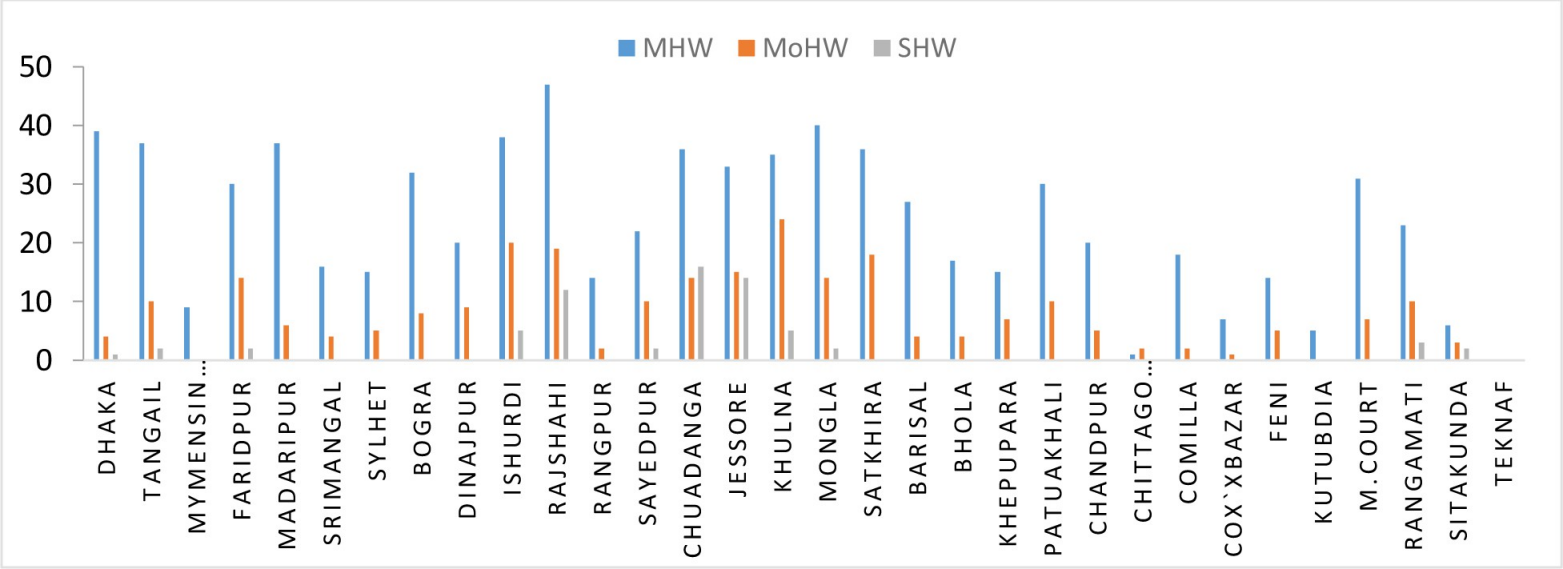
Seasonal frequency of HW days over all the stations for the period 2014.

As per the definition given by BMD, the heat wave is declared when Tmax≥36°с. In our study, we analyzed the Tmax≥36°с at 32 stations across Bangladesh for the period 1991–2021. It was found that the frequencies of days with Tmax≥36°с were maximum in most of the places over Bangladesh in 2014 (Fig 8). Most of the heat wave spells are found to prevail for many days and spread over more locations over the country in 2014. Out of 32 stations, 31 were struck by MHW in 2014, whereas MoHW hit 29 and SHW at 12 stations. In our research, it is found that the highest Tmax of 43.2°C was recorded at Chuadanga on 21 May 2014.

**Fig 9:**
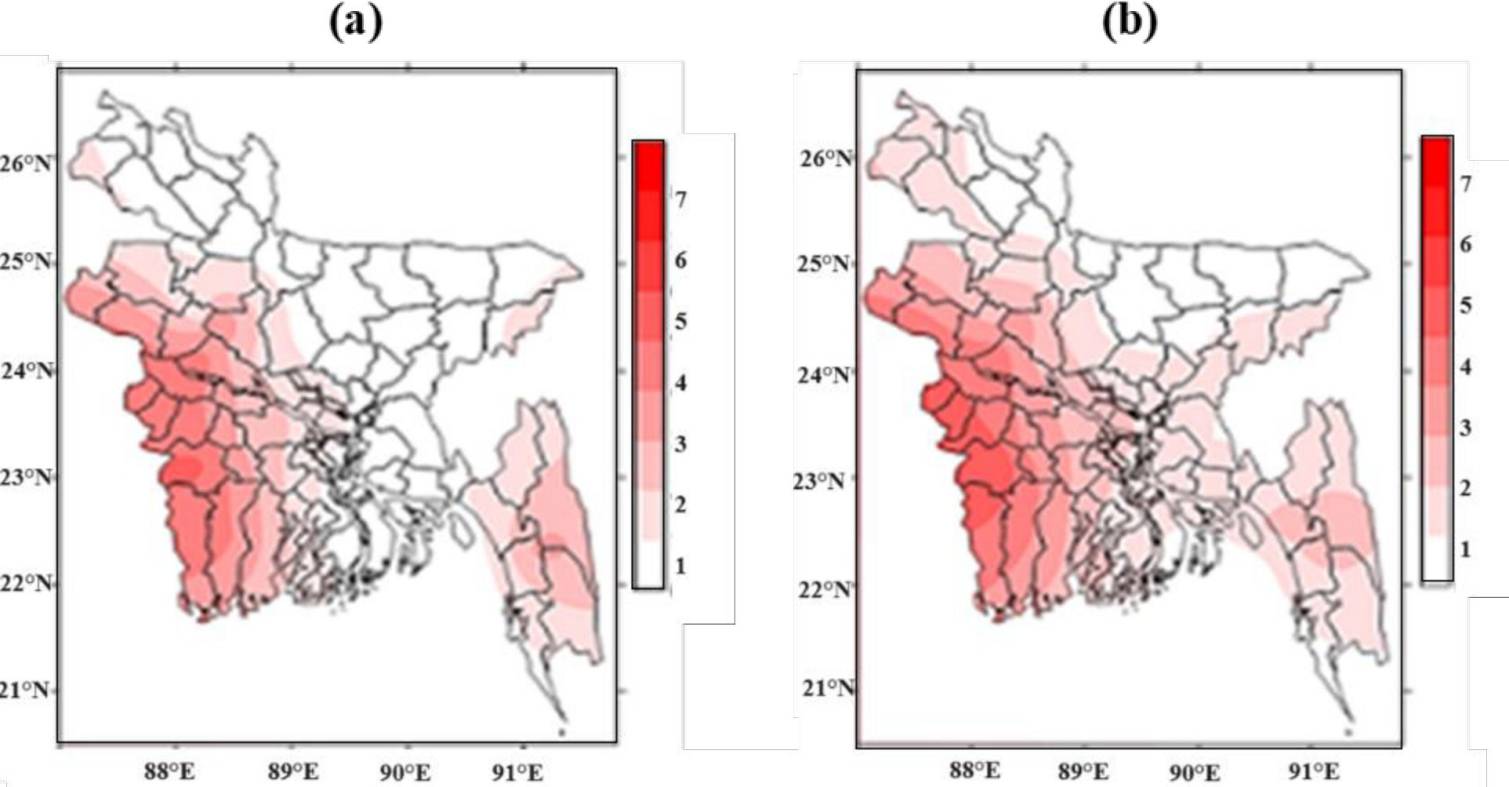
a) No. of HW (Apr 11–20) and b) No. of HW during Apr 21–30 in Bangladesh. The base map is an open-source shapefile from the NCEP/NCAR 40-year reanalysis dataset [45].

The spell of the heat wave was initiated over Khulna and Rajshahi divisions first. Then, it gradually spread over most parts of the country (Fig 9). After that the coverage of HW zone extended towards north and south over Khulna, Rajshahi and Dinajpur regions. Then, it extended towards east and covered the nearby area of its eastside but concentrated mainly over western part of Bangladesh till 20April 2014 (Fig 9A). During the narrated period, the severity reached its maximum criteria, and the highest maximum temperature was recorded 43.2°C at Jessore. Nearly all the stations recorded mild to severe heat wave situations throughout the country (Fig 9A). The strength of HW situation then weakened but concentrated over southwestern border regions and adjoining areas of Bangladesh. During 21–25 April it extended up to southeastern part of Bangladesh across central part and persisted as a moderate heat wave over this area, with its high focus over southwestern part (Fig 9B). Therefore, there were consecutive heat wave situation with high temperature and severe situation of heat wave persists over southwestern part of Bangladesh, which leads strong feelings of heat wave of this area. As a whole, the heat wave situation was terrible over southwestern part of Bangladesh and it was intolerable to the locality.

**Fig 10:**
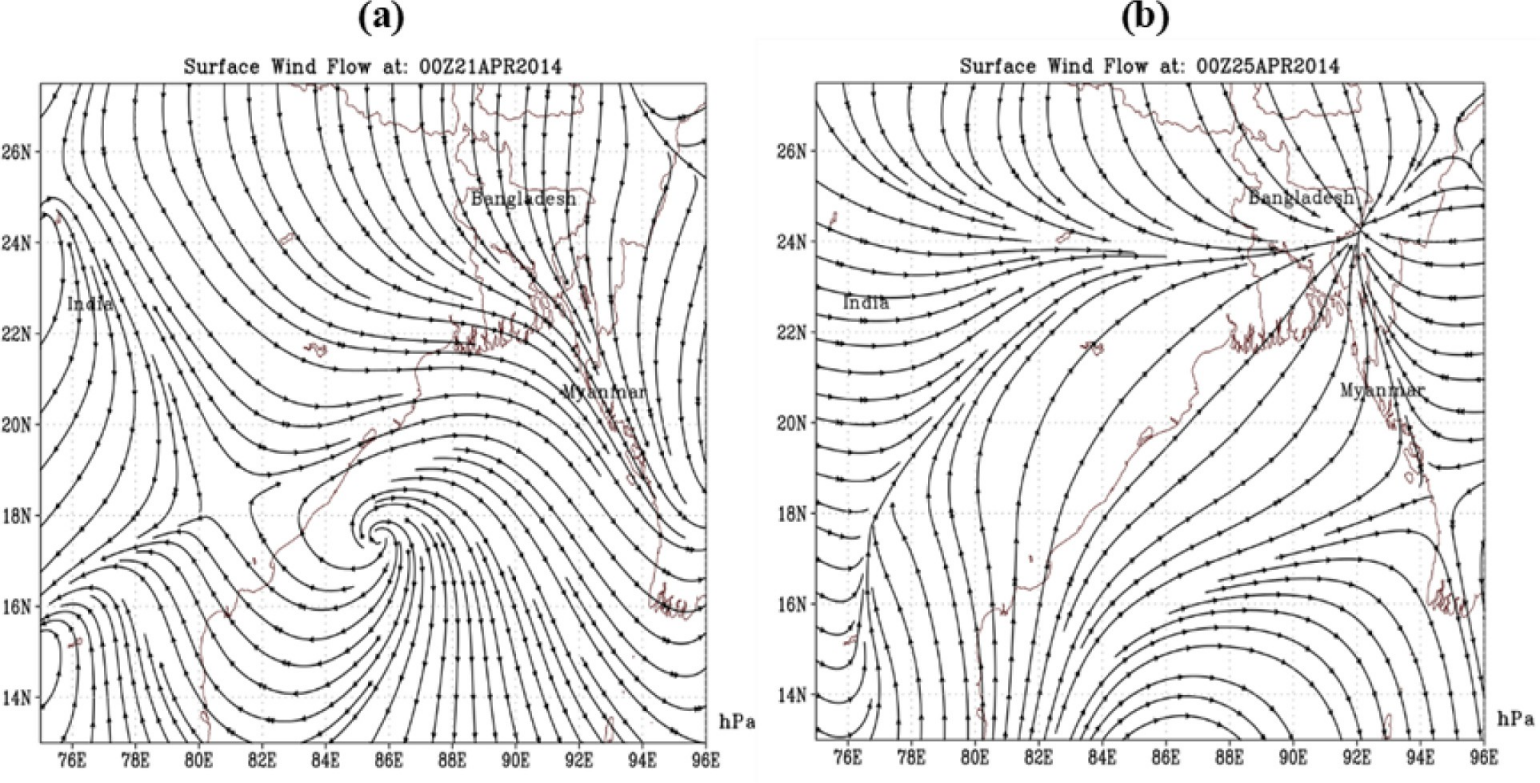
a) Surface wind on 21April, 2014 and b) Surface wind flow on 25April, 2014. The base map is an open-source shapefile from the NCEP/NCAR 40-year reanalysis dataset [45].

From the spatial distribution of surface wind 20^th^ April– 25^th^ April 2014, it is seen that strong dry westerly wind and weaker moist laden south westerly wind are observed and gradually a weak low-pressure system is experiencing over north western part of Bangladesh (Fig 10A and 10B).

### 3.8 Heat waves spell during 12–29 April 2021

**Fig 11:**
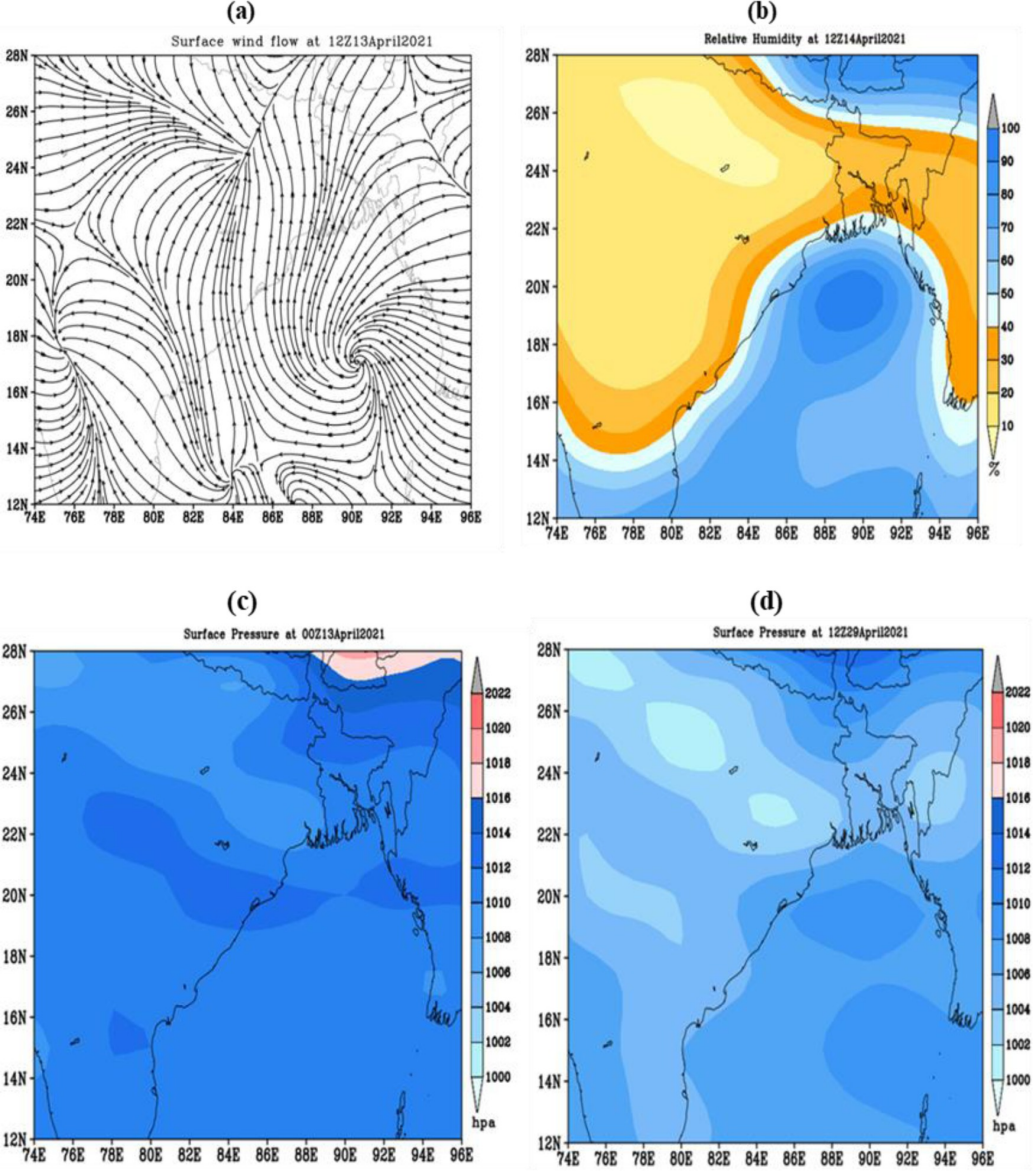
a) Surface wind on 13April, 2021 b) Spatial distribution of relative humidity on 14April, 2021 c) Surface pressure on 13April, 2021 and d) Surface pressure on 29April, 2021 over Bangladesh and adjoining area. The base map is an open-source shapefile from the NCEP/NCAR 40-year reanalysis dataset [45].

In 2021, heat waves throughout the country affected most of the stations and prevailed for a couple of days. During this duration, heat waves hit most parts of Bangladesh, starting over the Rajshahi region because of the persistence of a thermal low over Bihar and West Bengal that extended up to the southeastern part of the country. On 10 April, the heat wave initiated in this region, and then it spread out over Rajshahi division, Dhaka division, and most of the parts of Khulna division later. On 14 April 2021, the highest maximum temperature recorded was 39.7°C at Rajshahi. Due to an anticyclonic system at surface level over the central part of the Bay of Bengal, moisture flow was continuous BOB to the Bangladesh and adjoining area displayed in Fig 11A. This is why high humidity was present at the surface level during this period over Bangladesh; that is one of the reasons for heat waves (Fig 11B).

### 3.9 Heat wave event during 25–30 April 2021

**Fig 12:**
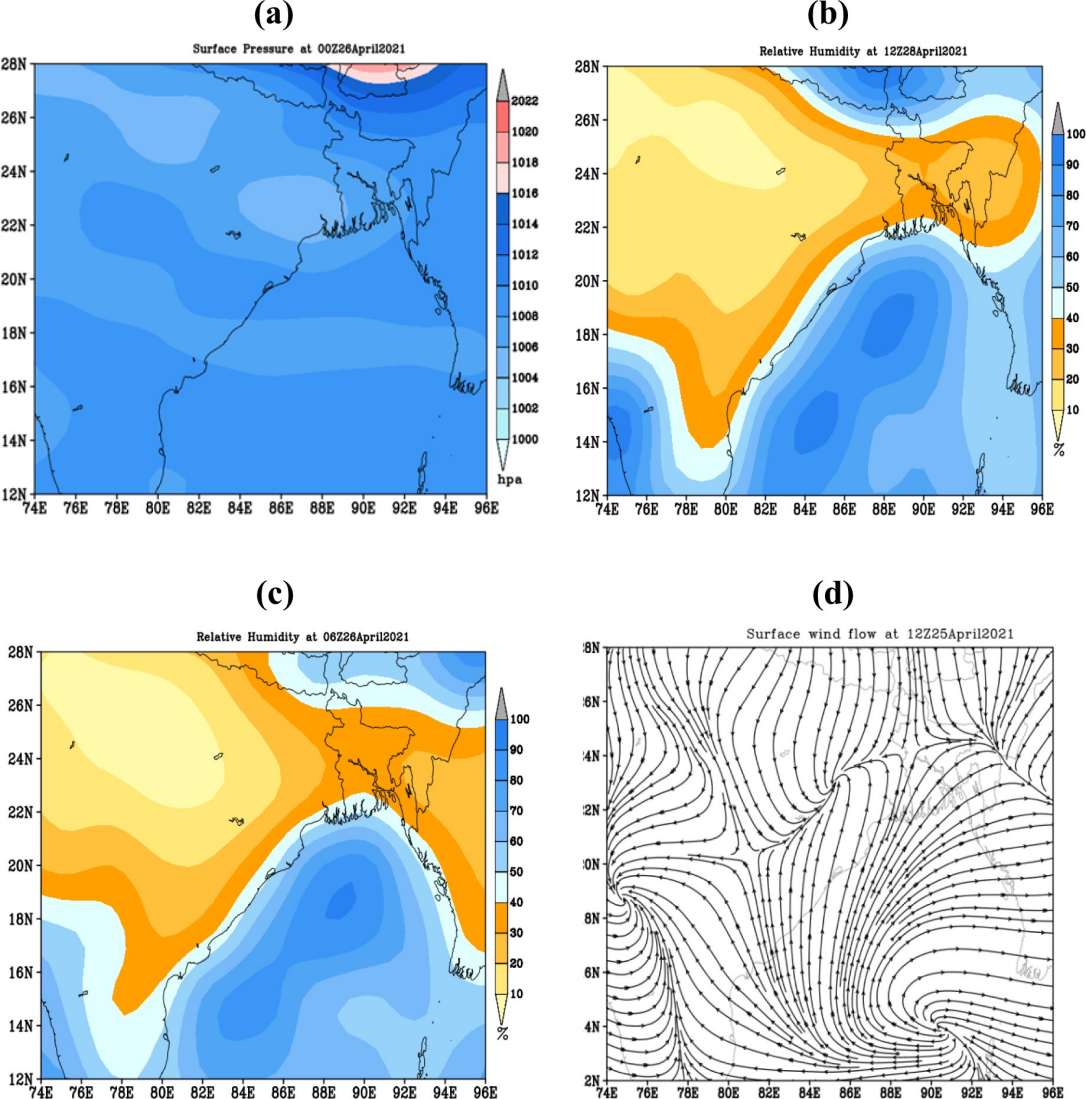
a) Surface pressure on 26 April, 2021 b) Relative humidity on 28April, 2021, c) Relative humidity on 26 April, 2021 and d) Surface wind on 25April, 2021. The base map is an open-source shapefile from the NCEP/NCAR 40-year reanalysis dataset [45].

Analysis of MSLP shows that a thermal low-pressure persistence over Bihar, West Bengal that extended up to the southeastern part of Bangladesh covers southern and central regions and has been displayed in **Fig 12.**

### 3.10 Spatial pattern of heat waves in Bangladesh

**Fig 13:**
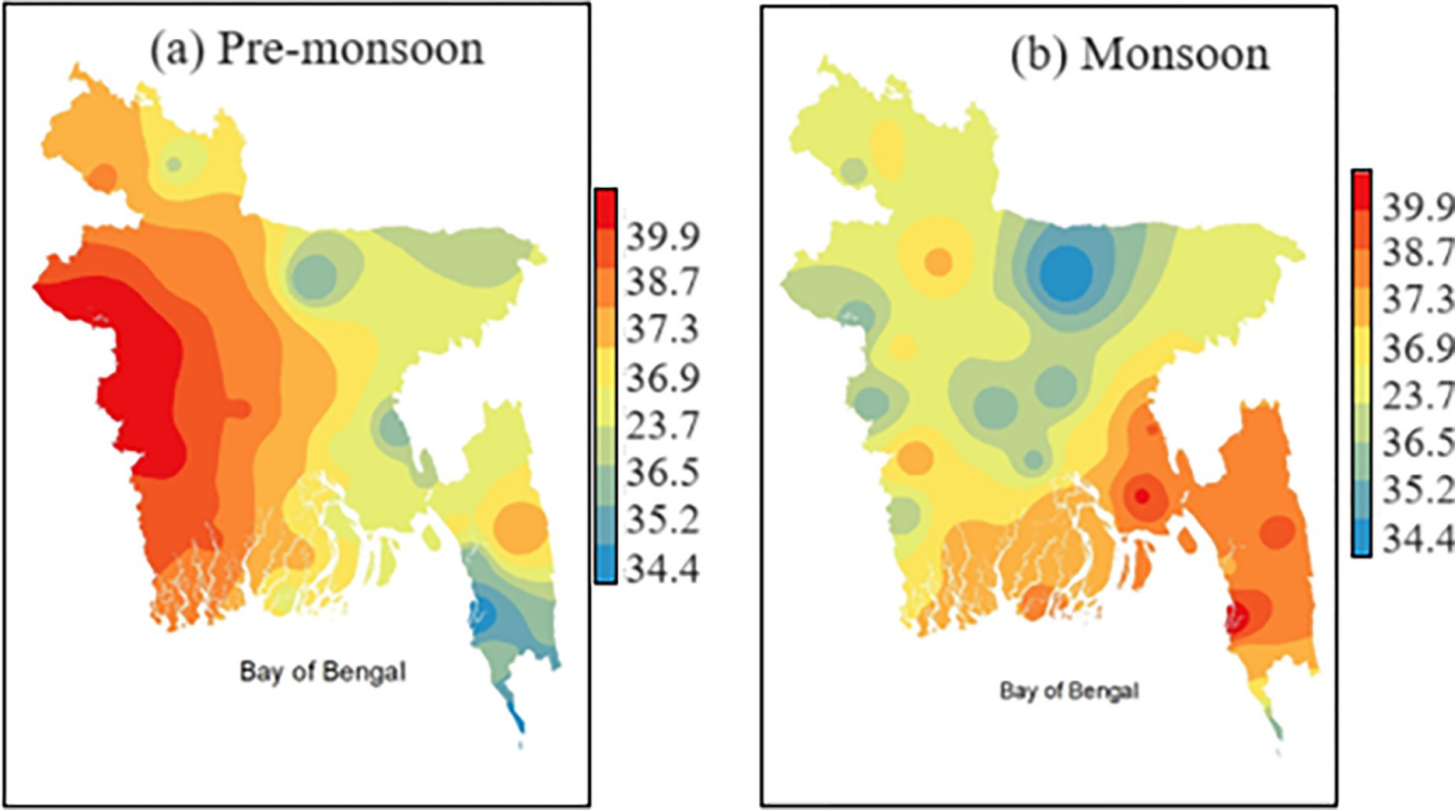
Spatial distribution of Tmax≥36°C during 1991–2021 in a) Pre-monsoon b) Monsoon (Author’s own map prepared by ArcGIS software).

The results reveal that heat waves are formed over central India that enter Bangladesh through the western part (Fig 13). During this time, the westerly and northwesterly winds become very strong, which causes the advection of warm air in Bangladesh. Due to the land heating generated by enormous solar energy and ample moisture intrusion, West Bengal and the adjoining areas of India and Bangladesh have become the hubs of the highest maximum temperature. Sometimes, it extended up to the southeastern part of Bangladesh. Fig 13 demonstrates the tendency to get heat waves, and the heat wave propagates towards southeast Bangladesh. The highest maximum temperature was recorded as 43.2°с at Chuadanga station on April 21, 2014. The second-highest maximum temperature was 42.6°C at Rajshahi on the same date. The maximum Tmax is found over the Chittagong Hill Tracts on the heat wave dates. In 2021, the highest maximum temperature was attained at 41.2°с at Jessore on April 25, 2021, and the second highest maximum temperature was found at Chuadanga, which was 40.5°с. The higher maximum temperature enters from the western part of Bangladesh and then gradually spreads towards the southeastern region. It is observed that the frequency of days is increasing in the western and southwestern parts of the country in the pre-monsoon season, while the southeast part experiences heat wave situations during the monsoon.

## 4. Discussion

Our research studied the heat wave conditions of different categories, namely MHW, MoHW, and SHW, during the pre-monsoon (March–May) and monsoon (June–September) for 30 years from 1991–2021. During the study period (1991–2021), Bangladesh recorded 1246 heat wave days with a Tmax≥36°C, °C, and 122 severe heatwave conditions. Since heat waves are a significant consequence of climate change [46], the study’s findings provide necessary information for understanding the impact of climate change on different aspects. It is noticed that heat wave conditions mainly affected the months of July, August, and September. Our analysis highlighted heat waves that form over central India and enter Bangladesh through the western part of the country. During the study period, the westerly and northwesterly winds became very prominent, which caused the advection of warm air into Bangladesh.

According to our data, a warmer temperature increases the frequency, severity, and duration of heat waves across the nation. Our findings are consistent with research showing a rise in heat waves nationwide [20, 32, 38]. There is also general agreement that heat waves will become longer period and more frequent. By thoroughly analyzing heat wave frequency and variability features and their potential causes of changes throughout time, this work contributes to the body of knowledge.

Lastly, Rahman et al. [20] and Nissan et al. [32] reported heat waves of an exceptional length of up to several months, consistent with our data that summers may consist of a single, extended heat wave. The anticipated rise in hot days is qualitatively consistent with [32], who presented data indicating an increase in the frequency of heat waves in Bangladesh, notwithstanding the difficulty of making a direct comparison. Future studies must investigate the reasons for this rise in heat persistence, as heat days’ persistence is a significant factor in how long heat waves last. Two possible reasons are the southeast trough getting shallower along with the northeast Indian thermal low [20] and the link between how long a heat wave lasts and soil moisture in a future climate with less rain [37]. Our findings demonstrate how sharply rising greenhouse gas concentrations might worsen Bangladesh’s heat-stress conditions.

Our results revealed increased MHW frequency and spatial distribution between 1991 and 2020, suggesting greater MHWs in recent decades due to the changing climate. During the study period, these MHWs were stimulated from mid-July to early August. Our analysis indicates that the warm surface air was probably responsible for Bangladesh’s rising MHW trends. Future research might concentrate on how large-scale atmospheric circulations are connected to the increasing tendencies of MHWs, as revealed by some related studies [47–49]. Studies on heat waves in various countries and regions, including Portugal [50], the Middle East [51], Iran [52], Northeast China [53], Central Europe [54], Bangladesh [9], and the Sahel [55], have shown positive trends that are consistent with a warming of earth’s climate [56].

The trend and variability of the frequencies of days with Tmax ≥ 36°C at the divisional stations of BMD have been studied from 1991–2021. The research shows that the seasonal (pre-monsoon) frequency of days with Tmax ≥ 36°C has an increasing trend at Dhaka, Chittagong, Barisal, Khulna, and Sylhet. During this period, the lowest frequency of days was found in Teknaf, Kutubdia, Mymensingh, and most coastal districts [46]. The study showed that the highest days of MHW were found in Rajshahi, 346, and the second highest days were found in Chuadanga, 287 days. The reason for this may be the advection of warm air from the western part of India and the geographical position of this region. Among the three decades, the last decade, D3 (2011–2021), was one of the most prominent decades, except in some cases due to the anomalies of atmospheric phenomena prevailing in the Indian subcontinent. In 2014, almost all the stations experienced heat waves throughout Bangladesh, significantly affecting the normal livelihood and other conditions of the geosystem. In this perspective, atmospheric conditions play an essential role. Among them, the presence of a high-pressure region, westerly/northwesterly wind flow, relative humidity, and lifting index are mostly mentionable. It can be said that there would be many options to work on the research topics in the near future. The projection of heat waves could be one of the future research topics mentioned above. It is noted that heat waves have become very susceptible to public health worldwide in the past few decades [57]. So, as a very alarming issue, the mortality rate due to heat waves can be explored further to a large extent. In 1996, the WMO prepared a vast review of the benefits of climatological information and services conducted by J.M. Nicholls (WMO/TD-No. 780). The study’s implications could be agriculture, livestock, fisheries, marine life, human health, social services, tourism, sports, disaster management, policy making, urban/town planning, and future studies. The topics chosen can also be of great assistance to climate researchers and managers all over the world. As a result of global warming, heat waves are anticipated to occur more often in the future. It’s also important to highlight the importance of natural climate variability since long-term changes in summer temperatures are likely linked to the sea surface temperature mode with a monopole structure over the Bay of Bengal [58].

Our analysis indicated that Bangladesh experiences heat waves. Due to the country’s extreme sensitivity, severe MHWs may imply a significant effect of heat waves on the country’s bio-ecosystem [59, 60]. The Bay of Bengal’s low seasonal fluctuation in sea surface temperature is related to the area’s high susceptibility. A heat early warning system must understand heat exposure and sensitivity. Extreme heat in Bangladesh, particularly in urban areas, must be shown to have human costs. However, this should not hinder policymakers from preventing heat-related deaths. High temperatures increase mortality in Bangladesh and other countries, especially among older adults, children, and males [61, 62]. Recent Indian Heat Early Warning Systems (HEWS) have proven that numerous common-sense actions may be adopted with minimal previous notice.

In our study, there are some limitations, such as the severe impacts of dangerous humid heat waves, as well as nighttime and compound heat waves, which have been mentioned in recent studies [32, 33, 48, 63]. The fact is that, due to some reasons, we have yet to consider them. Temporal discontinuities limit synchronous surface temperature readings at several sites. This is a significant constraint since the river and coastal winds are essential locally. To understand how natural and artificial landscapes affect urban and rural climates, a heat wave prediction model will be developed in a future study. Another limitation of the study is that this research did not consider the heat waves affecting population health research, which deserves further investigation. Some complex biometeorological indices, including Heat Index, Effective Temperature, and UTCI, do not consider other meteorological factors, such as wind, humidity, etc. Due to data inaccessibility, this research does not provide a quantitative examination of the relationship between the socioeconomic variables and the heat waves and duration of heat waves. This study did not consider heat waves and their changes caused by climate signals, including decadal climate variability (IOD, ENSO, MJO, BSISO), SST variability in the Bay of Bengal, etc. This paper investigates the role of atmospheric circulation patterns in determining heat wave conditions in the Indian subcontinent region [60]. In addition to this, an increase in evaporation and radiation heating caused by the acute anticyclonic anomaly strengthens a heat dome because of the positive water vapor feedback. The vast extent, including various large-scale atmospheric phenomena, geo-potential height, lifting index, surface wind at different levels, relative humidity, etc., should be investigated further. However, longer heatwaves due to global warming are expected to worsen the death burden in the northwest of the country in the future if mitigating efforts are not implemented.

## 5. Conclusion

This study intends to investigate the recent scenario and trend of the frequency of days of maximum temperature with various criteria, such as Tmax≥36°с, MHW 36–38°C, MoHW 38.1–40°C, and SHW 40–42°C, from 1991 to 2021. The present study also explored a possible cause of heat waves in Bangladesh. The results show that the frequencies of days with Tmax≥36°с have an increasing trend for seasonal and annual phenomena during 1991–2021 in Bangladesh, except for very few places. The study reveals that the country’s southwestern region has the highest increasing trend. The trend analysis shows a significant increasing trend of annual average day frequency (0.27 per year) across the country (p<0.05). In the pre-monsoon season, the frequency of days reached a maximum of 62 at the Chuadanga site. From 1991 to 2021, all of the stations under study experienced MHW except for Teknaf, while MoHW attacked all but three: Mymendingh, Kutubdia, and Teknaf, Rajshahi, Chuadanga, and Jessore sites are the three most affected stations by SHW. April and May are the two months in which the highest maximum temperature is recorded, and sometimes, the highest maximum temperature exceeds several times the average maximum temperature. The highest Tmax of 43.2°C was recorded at Chuadanga on May 21, 2014, which greatly concerns us. The trend of average days of frequency (Tmax≥36°C) is found to be high in the monsoon season (0.18 per year). Our study found that heat waves persist in the country for several days, enhancing the risk of diseases in various sectors. The increasing trend indicated a substantial rise in human hazards, drought events, fire risks, and energy demand in the northwest area in the past decade. The heat wave situation was terrible in southwestern Bangladesh, which was intolerable to the locality. Bangladesh experiences heat waves and heat wave spells due to the warm air advection from the west or southwest part of the country. Heat waves move towards the eastern part of Bangladesh and then extend up to the central part of the country. It is alarming that the changing climate pattern is accelerating the intensity and severity of heat waves. Due to the availability of adequate moisture, the prominent influence of subtropical highs over India and their extension contributed to generating heat waves across Bangladesh at the surface level. In the context of global warming, these tendencies are alarming. These results may help guide the development of preventative strategies to lessen the adverse effects of heatwaves on the ecosystem and public health.

## Supporting information

S1 Data (RAR)

## References

[1] IPCC, 2018.Summary for policymakers. In: Masson-Delmotte V., et al., (Eds.), Global Warming of 1.5°C. An IPCC Special Report on the Impacts of Global Warming of 1.5°C above Preindustrial Levels and Related Global Greenhouse Gas Emission Pathways, in the Context of Strengthening the Global Response to the Threat of Climate Change, Sustainable Development, and Efforts to Eradicate Poverty. World Meteorological Organization, Geneva, Switzerland, p. 32.

[2] Wolf T., McGregor G., 2013.The development of a heat wave vulnerability index for London, United Kingdom.Weather Clim.Extrem. 1, 59–68. 10.1016/j.wace.2013.07.004.

[3] Zander K., Botzen W., Oppermann E., Kjellstorm T., Garnett S.T., 208. Heat stress causes substantial labour productivity loss in Australia. Nat. Clim. Change 5, 647–651

[4] Guo Y., Gasparrini A., et al., 2017. Heat wave and mortality: a multicountry, multicommunity study. Environ. Health Perspect. 125 doi: 10.1289/EHP1026 28886602 PMC5783630

[5] Liss A., Wu R., Chui K., Naumova E., 2017. Heat-related hospitalizations in older adults: an amplified effect of the first seasonal heatwave. Sci. Rep. 7, 39581 doi: 10.1038/srep39581 28074921 PMC5225426

[6] Roy´e D., Codesido R., Tobías A., Taracido M., 2020.Heat wave intensity and daily mortality in four of the largest cities of Spain. Environ. Res. 182, 09027. doi: 10.1016/j.envres.2019.109027 31884190

[7] Campbell S., Remenyi T.A., White C.J., Johnston F.H., (2018) Heatwave and health impact research: A global review, Health & Place, 53: 210–218, doi: 10.1016/j.healthplace.2018.08.017 30189362

[8] Raei E., Nikoo M.R., AghaKouchak A., Mazdiyasni O., Sadegh M., 2018.GHWR, a multi‒method global heatwave and warm‒spell record and toolbox.Sci. Data 5, 180206. 10.1038/sdata.2018.206 PMC6207177 30376556

[9] Nissan H., Burkat K., de Prez E. C., Aalst M.V., and Mason S., 2017. Defining and Predicting Heat Waves in Bangladesh, Journal of Applied Meteorology, 56, 2653–2670.

[10] Lubna K.F., Mallik M.A.K., Alam M.S., Jimmy A.N., Islam T., Ahmad I., et al. 2020. Study on Heat Wave and its Thermodynamic features over Bangladesh using Numerical Weather Prediction Model (NWPM). International Journal of Science and Business, 4(6), 44–52. 10.5281/zenodo.3839997.

[11] Keatinge W R, Donaldson G D, Cordioli E, Martinelli M, Kunst A E, et al., 2000: Heat related mortality in warm and cold regions of Europe: observational study. Br Med J 321: 670–673. doi: 10.1136/bmj.321.7262.670 10987770 PMC27480

[12] Guest C S, Wilson K, Woodward A, Hennessy K, Kalkstein L S, et al., 1999: Climate and mortality in Australia: retrospective study, 1979–1990 and predicted impacts in five major cities in 2030. Clim Res 13:1–8. 13. Volume 8 MANNAN ET AL. March 2022160

[13] Kumar S, 1998: Indian heat wave and rains results in massive death toll. Lancet 351:1869.

[14] Pan W H and Li L A, 1995: Temperature extremes and mortality from coronary heart disease and cerebral infarction in elderly Chinese. Lancet, 345,353–356. doi: 10.1016/s0140-6736(95)90341-0 7845116

[15] UNISDR, USAID, and Centre for Research on the Epidemiology of Disasters, 208: 208 disasters in numbers. Infographic, 2 pp., http://www.unisdr.org/files/47804_208disastertrendsinfographic.pdf.

[16] Huq S., 1974: Climate of Bangladesh. Studies in Bangladesh Geography, Kamaluddin A. F. M, Ed., Bangladesh National Geographical Association, 3.

[17] Karmakar S., 2019. Patterns of climate change and its impacts in northwestern Bangladesh. Journal of Engineering science, 10(20), 33–48.

[18] Rajib M.A., Mortuza M.R., Selmi S., Ankur A.K., Rahman M.M., 2011. Increase of heat index over Bangladesh: impact of climate change. World Acad. Sci. Eng. Technol. 58, 402–405.

[19] Al-Maruf A., 2017.Enhancing disaster resilience through human capital: prospects for adaptation to cyclones in coastal Bangladesh (Doctoral dissertation).Universitat ¨ zu Koln¨.

[20] Rahman M B, Salam R, Islam A,R,M,T, Tasnuva A, Haque U., Shahid S., et al. 2021 Appraising the historical and projected spatiotemporal changes in the heat index in Bangladesh, Theoretical and Applied climatology, doi: 10.1007/s00704-021-03705-x 34334853 PMC8302469

[21] Ratnam J.V., Behera S.K.1, Ratna S. B., Rajeevan M., and Yamagata T., 2016. Anatomy of Indian heat waves, Scientific Reports | 6:24395 | doi: 10.1038/srep24395 1 www.nature.com/scientificreports, pp. 1–11. 27079921 PMC4832141

[22] Pai D.S., Nair S.A., and Ramanathan A.N., 2013. Long term climatology and trends of heat waves over India during the recent 50 years (1961–2010), Mausam, 64(4), 585–604.

[23] Mishra V., Ganguly A. R., Nijssen B., and Lettenmaier D. P., 208. Changes in observed climate extremes in global urban areas, Environ. Res. Lett. 10, 2, 024005 doi: 10.1088/1748-9326/10/2/024005

[24] Panda D. K., Agha Kouchak A., and Ambast S. K., 2017. Increasing heat waves and warm spells in India, observed from a multiaspect framework, Journal Geographical Research: Atmosphere, 122(7), 1–22.

[25] Mitra V., Mukherjee S., Kumar R., and Stone D. A., 2017. Heat wave exposure in India in current, 1.5°C, and 2.0°C worlds, Environ. Res. Lett., 12, 124012, 1–9, 10.1088/1748-9326/aa9388.36204013

[26] De U. S., and Mukhopadhyay R. K., 1998. Severe heat wave over Indian subcontinent in 1998 in a perspective of global climate, Current Science, 75, 1308–1311.

[27] Murari K., Ghosh S., Patwardhan A., Daly E., and Salvi K., 208. Intensification of future severe heat waves in India and their effect on heat stress and mortality.Regional Environmental Change, 8, pp.569–57.

[28] Ghatak D., Zaitchik B., Hain C., and Anderson M., 2017. The role of local heating in the 208 Indian Heat Wave, Sci Rep 7, 7707. 10.1038/s4898-017-07956-5. 28794447 PMC5550505

[29] Mishra V., Ganguly A. R., Nijssen B., and Lettenmaier D. P., 208. Changes in observed climate extremes in global urban areas, Environ. Res. Lett. 10, 2, 024005 doi: 10.1088/1748-9326/10/2/024005

[30] Ahmed R., and Karmakar S., 1993: Arrival and withdrawal dates of the summer monsoon in Bangladesh. Int. J. Climatol., 13, 727–740, doi: 10.1002/joc.3370130703

[31] IPCC, 2014. Climate change 2014: synthesis report. In: Core Writing Team, Pachauri R. K., Meyer L.A. (Eds.), Contribution of Working Groups I, II and III to the Fifth Assessment Report of the Intergovernmental Panel on Climate Change. IPCC, Geneva, Switzerland, 81 pp.

[32] Nissan H., Muñoz A.G., Mason S.J., 2020, Targeted model evaluations for climate services: A case study on heat waves in Bangladesh, Climate Risk Management, 28, 10.1016/j.crm.2020.100213.

[33] Tawsif S., Alam M.S, Al-Maruf A. 2022, How households adapt to heat wave for livable habitat? A case of medium-sized city in Bangladesh, Current Research in Environmental Sustainability,10089, 4, 10.1016/j.crsust.2022.10089.

[34] Imran H.M., Kala J., Uddin S., Islam A.K.M.S., Acharya N., 2023, Spatiotemporal analysis of temperature and precipitation extremes over Bangladesh using a novel gridded observational dataset, Weather and Climate Extremes, 39, 10.1016/j.wace.2022.100544.

[35] Dewan A., Kiselev G., Botje D., Mahmud G.I., Bhuian M.H., Hassan Q.K., 2021, Surface urban heat island intensity in five major cities of Bangladesh: Patterns, drivers and trends, Sustainable Cities and Society, 71, 10.1016/j.scs.2021.102926.

[36] Wedler M, Pinto J.G., Hochman A., 2023. More frequent, persistent, and deadly heat waves in the 21st century over the Eastern Mediterranean, Science of The Total Environment, 870, 161883 doi: 10.1016/j.scitotenv.2023.161883 36736407

[37] Rahman M.S., Islam A.R.M.T., 2019. Are precipitation concentration and intensity changing in Bangladesh overtimes? Analysis of the possible causes of changes in precipitation systems, Science of the Total Environment, 7.96, 690:370–387, doi: 10.1016/j.scitotenv.2019.06.529 31299571

[38] Huq S., 2001: Climate change and Bangladesh. Science, 294, doi: 10.1126/science.294.5547.1617 11721020

[39] Ashraf S.A., Faruk M. 2018, Children’s perspective on adaptation to heat waves and heavy precipitation in Dhaka, Bangladesh, Procedia Engineering,212, 768–775, 10.1016/j.proeng.2018.01.099.

[40] Bhattacharjee A., Hassan S.M.Q., Hazra P., Kormoker T., Islam S., Alam E., et al (2023) Future Changes of Summer Monsoon Rainfall and Temperature Over Bangladesh Using 27 CMIP6 Models, Geocarto International, doi: 10.1080/10106049.2023.2285342

[41] Mahmud Khandakar & Soshantabinteabid, & Ahmed Raju. (2018). Development of Climate Classification Map for Bangladesh Based on Koppen’s Climate Classification.

[42] Sanderson M., and Ahmed R., 1979: Pre-monsoon rainfall and its variability in Bangladesh: A trend surface analysis. Hydrol. Sci. Bull., 24, 277–287, doi: 10.1080/02626667909491867

[43] Mostafiz & Shajib, 2021: The Behavior of climatic parameters over Rangpur division, Dew Drop, 7(1), 145–152.

[44] Mora C., et al. 2017. Global risk of deadly heat Nat. Clim. Chang., 7 501–506, doi: 10.1038/nclimate3322

[45] Kalnay et al., The NCEP/NCAR 40-year reanalysis project, Bull. Amer. Meteor. Soc., 77, 437–470, 1996

[46] Ghose B, Islam ARMT, Kamruzzaman M, Moniruzzaman M, Hu Z (2021) Climate induced rice yield anomalies linked to large-scale atmospheric circulation in Bangladesh using multi-statistical modeling, Theoretical and Applied climatology, TheorApplClimatol 144, 1077–1099.

[47] Wang J., Chen Y., Liao W., He G., Tett S.F.B., Yan Z., et al., 2021. Anthropogenic emissions and urbanization increase risk of compound hot extremes in cities. Nature.Clim. Change 11 (12), 1084–1089.

[48] Huang B., Wang Z., Yin X., Arguez A., Graham G., Liu C., et al. (2021). Prolonged marine heatwaves in the Arctic: 1982−2020. Geophysical Research Letters, 48, e2021GL095590. 10.1029/2021GL095590.

[49] Oliveira A.; Lopes A.; Correia E.; Niza S.; Soares A. Heatwaves and Summer Urban Heat Islands: A Daily Cycle Approach to Unveil the Urban Thermal Signal Changes in Lisbon, Portugal. Atmosphere 2021, 12, 292.

[50] Zittis G., Hadjinicolaou P., Fnais M., Lelieveld J., (208) Projected changes in heat wave characteristics in the eastern Mediterranean and the Middle East, Reg Environ Change, doi: 10.1007/s10113-014-0753-2

[51] Safieh J., Rebwar D., Forough J. (2020) Effect of climate change on heat waves in the South Sea of Iran, Ukrainian Journal of Ecology, 2020, 10(5), 87–93.

[52] Kysel´y J. (2010) Recent severe heat waves in central Europe: how to view them in a long-term prospect? Int. J. Climatol. 30: 89–109.

[53] Oliver E. C. J., Donat M. G., Burrows M. T., Moore P. J., Smale D. A., Alexander L. V., et al. (2018). Longer and more frequent marine heatwaves over the past century.Nature Communications, 9, 1324. doi: 10.1038/s41467-018-03732-9 29636482 PMC5893591

[54] Sambou M.G., Pohl B., Janicot S., Famien A.M.L., Roucou P., Badiane D., et al. (2018) Heat waves in spring from Senegal to Sahel: Evolution under climate change, Int. J. Climatol. doi: 10.1002/joc.7176

[55] Mallick J, Islam ARMT et al. (2022) Spatiotemporal trends of temperature extremes in Bangladesh under changing climate using multi-statistical techniques, Theoretical and Applied climatology, doi: 10.1007/s00704-021- 03828–1.

[56] Aströ m DO, Forsberg B, Rocklö v J. Heat wave impact on mor-bidity and mortality in the elderly population: a review of recentstudies. Maturitas 2011; 69:99–105. doi: 10.1016/j.maturitas.2011.03.008 21477954

[57] Islam HMT, Islam ARMT, Abdulllah-al-mahbub M, Shahid S, Tasnuva A, Kamruzzaman M, et al (2021) Spatiotemporal changes and modulations of extreme climatic indices in monsoon-dominated climate region linkage with large-scale atmospheric oscillation, Atmospheric Research, 264, 105840, doi: 10.1016/j.atmosres.2021.105840

[58] Burkart K., Breitner S., Schneider A., Khan M. H., Kramer A., and Endlicher W., 2014a: An analysis of heat effects in different subpopulations of Bangladesh. Int. J. Biometeor., 58, 227–237, doi: 10.1007/s00484-013-0668-5 23689928

[59] Rogachev K., &Shlyk N. (2021).Record-breaking warming in the Kamchatka Current halocline. Ocean Dynamics, 71(5), 545–557.

[60] Burkart K., and Endlicher W., 2011: Human bioclimate and thermal stress in the megacity of Dhaka, Bangladesh: Application and evaluation of thermophysiological indices. Health in Megacities and Urban Areas, Krämer A, Khan M. H, and Kraas F, Eds., 83–170.

[61] Yu S, TETT S. F. B, FREYCHET N, YAN Z. W, 2021, Changes in regional wet heatwave in Eurasia during summer (1979–2017). Environmental Research Letters, 16.

[62] Wang J., Feng J.M., Yan Z.W., Chen Y., 2020. Future risks of unprecedented compound heat waves over three vast urban agglomerations in china. Earths. Future 8.

[63] Baldwin J.W., Dessy J.B., Vecchi G.A., Oppenheimer M., 2019. Temporally compound heat wave events and global warming: an emerging hazard. Earths Future 7, 411–427.

